# Structural controls on bedrock weathering in crystalline basement terranes and its implications on groundwater resources

**DOI:** 10.1038/s41598-022-15889-x

**Published:** 2022-07-12

**Authors:** Rudra Mohan Pradhan, Anand Singh, Arun Kumar Ojha, Tapas Kumar Biswal

**Affiliations:** 1 Department of Earth Sciences, Indian Institute of Technology Bombay, Powai, 400 076 India; 2 National Geophysical Research Institute, Hyderabad, 500 007 India

**Keywords:** Hydrogeology, Geophysics

## Abstract

Crystalline basement rock aquifers underlie more than 20% of the earth’s surface. However, owing to an inadequate understanding of geological structures, it is challenging to locate the groundwater resources in crystalline hard rock terranes. In these terranes, faults, fractures, and shear zones play an important role in bedrock weathering and ultimately groundwater storage. This study integrates important geological structures with 2D high-resolution subsurface resistivity images in understanding the factors that influenced bedrock weathering and groundwater. The results reveal the variability of weathered zone depth in different structural zones (Zone-I to Zone-IV). This is due to the presence of foliations, fractures, and faults. A thicker weathered zone develops when a fracture/fault overprints a pre-existing planar pervasive structure like foliations (Zone-II) as compared to zones only with faults/fractures (Zone-III). Further, the transmissivity of boreholes also shows relatively higher in Zone-II than Zone-III, which implies a good pact between different structural features and possible groundwater storage. The study also demonstrates the role of paleostress and different tectonic structures influencing the depth of the “Critical Zone”. While the geology may vary for different structural terranes, the approach presented in this paper can be readily adopted in mapping bedrock weathering and groundwater resources in crystalline basement terranes globally.

## Introduction

Globally, crystalline basement rock aquifers underlie more than 20% of the total earth’s surface^1–6^ however, they are less understood than soft (porous) rock aquifers. This is because of their heterogeneous nature on a local to regional to continental scale, which makes it challenging to quantify and compare them with other similar aquifers^7,8^. The crystalline rocks which occupy a major portion of the earth’s crust are devoid of primary porosity^9^. Several authors used secondary structures such as structural lineaments for mapping groundwater potentials/recharge zones, especially in arid- and semi-arid crystalline basement terranes^7,10–15^. Since the large-scale lineaments have been collected for regional groundwater studies, it is difficult to cross-check every tectonic feature in the field. On the other hand, the near-surface electrical resistivity method is a widely used geophysical tool over structural discontinuities for groundwater exploration which helps in determining the aquifer thickness, fractured zones, and depth to bedrock^16,17^. Nowadays, 2D and 3D resistivity imaging techniques are being used over 1D resistivity surveys for mapping subsurface features^18–21^. This method is also used to understand the subsurface flow of contaminated groundwater based on the resistivity contrasts^19,22^.

Solomon and Ghebreab^13^ used remote sensing and geographic information system-based lineament analysis for understanding the hydrotectonics of the crystalline terrane. Neves and Morales^23^ focused on the structural features that control the hydraulic properties of hard rock aquifers in southeastern Brazil, whereas Place et al.^24^ used electrical resistivity imaging, seismic tomography, and ground penetrating radar to understand the fault zones and their controls on the weathering process in the Catalan Coastal Ranges, NE Spain. However, most of the study has not attempted in detail structural analysis of brittle-ductile zones and their role in weathering processes and groundwater resources. In these terranes, the tectonic structures play an important role in understanding the deep-seated aquifer systems. Though the role of the fracture parameters viz. orientation, density, spacing, and continuity can be determined based on numerical and conceptual models^25^, the main effects on groundwater occurrence and availability in the field are still not understood properly. Further, the groundwater in crystalline basement terranes is multivariate due to different controlling factors i.e., topography, bedrock type, weathered thickness, and their extent, size, and orientation of faults and fracture networks^26,27^. The faults and fracture networks which are the primary source of water supply in these terranes developed due to crustal stresses caused during various tectonic events, cooling of magma, change of temperature, and release of overburden^28^. The different tectonic structures in the rocks such as foliations, lineations, folds, faults, fractures, and joints, developed during plastic-elastic flowage under dynamo-thermal metamorphic conditions. During the long period of geological history, these rocks have undergone deformation and tectonised in many ways that dictate the secondary porosity of the formation. Besides, the weathering process which is active continuously has either increased or decreased the secondary porosity depending on the rock types, tectonics, and climatic conditions^29,30^. Moreover, the fault zones may serve as barriers, conduits, or a combined conduit-barrier structure, depending upon the architecture of the fault rock materials^7,9,24^.

It is quite evident that both structural geologists and hydrogeologists (including geophysicists) often overlook each other while dealing with groundwater resources, especially in hard rock terranes. Structural entities include brittle zones, brittle-ductile zones, and ductile shear zones in these deformed granitic terranes. Further, the regional stress condition (paleostress) of an area is also quite important in explaining the tectonic processes and their role in bedrock weathering and groundwater storage.

In hard rock terranes, the groundwater is mainly confined to the local weathered zones^15,31,32^. The reactive transport model envisages that the propagation of the weathered zone depends on the balance between the groundwater residence time and mineral reaction kinetics^30^. It further suggests that the fluid flow path controlled by the fracture systems also regulates the downward propagation of the weathered zone. For this reason, the bedrock fracture system is considered to be the utmost notable factor that facilitates weathering and hence the weathered zone. Tectonic fractures or shear zones controlled by tectonic stress manage the groundwater in different areas around the globe^7,13,23,24,33^. In contrast to this view, another school of thought is that only the weathering process controls fracture permeability in hard rock terranes; neither tectonic stress nor the unloading has any control on fracture permeability^6,34^. However, several questions persist, particularly in the hard rock terranes are:

(i) *What is the role of pervasive structures like cleavage and foliations on weathered zone thickness and groundwater resources?* Pervasive features like foliations and cleavages are common in tectonically deformed terranes. As these pervasive features are the planes of weakness in rocks, they must have some degree of control in the weathered zone development process and groundwater.
(ii) *What is the role of multi-deformation events on bedrock weathering and groundwater storage?* Multi-episodes of deformation in a tectonically deformed terrane are common because of ongoing plate tectonics since the inception in the Mesoarchean era. Different episodes of deformation generate a set of deformation structures and increase the fault/fracture densities that can facilitate the groundwater in the subsurface. However, how the different tectonic structures related to different episodes of deformation interact and manage the groundwater resources needs more detailed studies.

Finding these gaps, this study is designed to explore the role of pervasive structures and different deformation events for groundwater in hard rock terranes. We address this by considering the Ambaji regions of South Delhi Terrane (SDT) as a case study, as the study region exposes high-grade metamorphic and crystalline rocks. The SDT is a part of the Aravalli-Delhi Mobile Belt (ADMB), NW India. The area is deliberately considered because (i) the area mainly exposes hard rocks, (ii) well-constrained geology and geological events, and (iii) the area suffers acute groundwater shortage and over-exploitation of aquifers for a long time due to the semi-arid climate.

Topography stress is one of the critical factors that control the weathered zone thickness in the “Critical Zone”^30^. The Critical Zone is an important life sustaining earth’s layer which extends from the subsurface depths where groundwater freely circulates to the top of the tree canopy including weathered bedrocks and soil^30^. In fact, the role of topographic stress will be high in rugged terrain with relatively weak rocks with tectonic stress. St. Clair et al.^30^ presented a detailed analysis of the role of topography on weathered zone thickness and its implication on the presence of regional tectonic stress. However, there is a clear-cut gap in the understanding of the role of paleostress and structure in weathered zone depth and its role in the Critical Zone. The present study explores this scientific gap using structural geology integrated with high-resolution 2D Electrical resistivity imaging. In the present study, we are neglecting the role of topographic stress because of the following reasons i.e., (a) the area is tectonically inactive. Therefore, the topography will perturbate the gravitational stress only^30^ and (b) the area is not subjected to intense topographic perturbation. Hence, the effect of topography can be neglected.

## Study area

**Figure 1:**
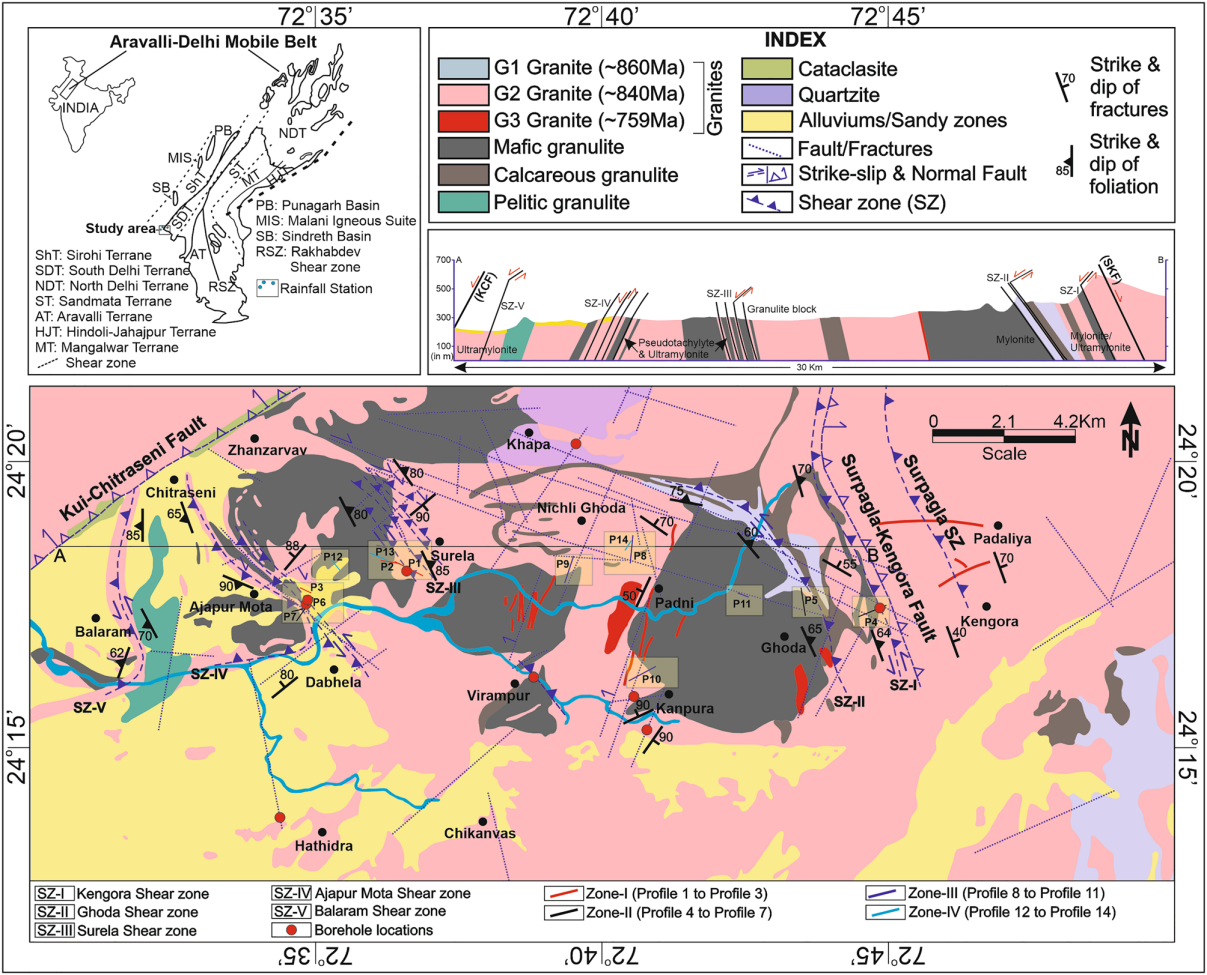
Geological and structural map of the study area with major shear zones, fracture, faults, and litho-units. Inset shows the Aravalli-Delhi Mobile belt (ADMB) and associated terranes. The square box (shaded yellow) shows the geophysical survey sites (P1-P14).

The study area (Ambaji basin) covers approximately 560 km^2^ and is located in the southern part of the ADMB, NW India. The area lies between latitude 72°30′–72°50′ N and longitude 24°10′–24°22′ E (Fig. 1). It is characterized by a semi-arid climate with different seasons i.e., extremely hot during summer (March to July), dryness in winter (December to January), and scanty rainfall during the rainy season (July to September). The elevation ranges from 215 to 810 m above Mean Sea Level (MSL). The average temperature ranges from 35 to 42 °C and the annual average precipitation is 805 mm mostly received through the southwest monsoon^15,35,36^.

### Geological settings

The study area comes under South Delhi Terrane (ca. 1100–900 Ma) of the ADMB, NW India. The ADMB comprises several terranes that are accreted to the mobile belt along the shear zones through various orogenic cycles (Fig. 1 inset)^37^. Further, the ADMB witnessed several granitic intrusions that have occurred in different ages such as the Ambaji granite, Berach granite, the Sendra granite, Mount Abu granite, and the Erinpura granite. Most of the granitic intrusions are confined in the northwestern and southwestern parts of the ADMB. The ADMB consists of predominantly low-grade metamorphic rocks except for a few patches of granulites such as Ambaji granulite (study area), Sandmata granulite, and Pilwa-Chinwali granulites^37^.

The Ambaji granulite has multiply deformed at different stages and subsequently uplifted through the exhumation process from the lower-middle earth crust^37,38^. The granulite has intruded by three distinct granites namely G1 (gneissic structure), G2 (coarse-grained) which is found to be predominant in the study area, and G3 (fine to medium-grained occurs as a vein). In contrast to these granitic rocks, the other medium and high-grade rocks in the Ambaji basin are comprised of quartzite, mafic- and calcareous-granulite. The ages of three different granites G1, G2, and G3 are 860 Ma, 840 Ma, and 759 Ma respectively^37^. The rocks in this region are highly fractured and both large-scale brittle- and ductile shear zones have been developed due to multiple deformation events. Further, the area is bounded by two major faults i.e., the Surpagla-Kengora fault in the east and the Kui-Chitraseni fault in the west (Fig. 1). The central part was thrusted up, and later extension and strike-slip movement occurred at different events (Fig. 1). This has produced numerous normal faults and shear zones. There are major and minor structural lineaments (fractures/faults) and six major shear zones (SZ) such as Surpagla SZ, Kengora SZ, Ghoda SZ, Surela SZ, Ajapur Mota SZ, and Balaram SZ (mostly NW–SE and NNW-SSE trending) present within the study basin (Fig. 1). Recent alluviums and sand dunes are found in the southwest part of the study area.

## Materials and methods

### Structural mapping and data collection

Extensive field studies that include attitude data collections of foliations, fractures, and fault-slip measurements for paleostress estimations were conducted using standard and well-established procedures. Along with this, lithological contacts and shear zones were mapped. Rock samples were also collected for microstructural analysis under a petrological microscope.

### Paleostress analysis

The brittle tectonic stress analysis is commonly based on observations from fault-slip data collected from the outcrops. A brief outline of the fault-slip analysis methods is presented here as this method is well established. Three basic assumptions that laid the foundation of the paleostress estimation from fault-slip analysis methods are as follows:

- The movement on one fault is independent of other faults, and the bulk stress state is homogeneous^39,40^.
- Slip always takes place along the direction parallel to the slicken fibers that represent the maximum resolved shear stress on the plane of movement under a given state of stress (Wallace-Bott Hypothesis)^41,42^.
- All the faults are homogeneous and part of the same tectonic event^40,43–45^.

Since the deduction of all the stress tensor components is impossible from the fault-slip data, the paleostress inversion aims to deduce a reduced stress tensor: the orientation of the principal stress axes σ_1_, σ_2_ and σ_3_ and the stress ratio (R), and the ratio of the principal stress magnitude difference.

1 $$\mathrm{R}= \frac{\left({\sigma }_{2}-{\sigma }_{3}\right)}{\left({\sigma }_{1}-{\sigma }_{3}\right)}$$

R defines the shape of the stress ellipsoid. The stress regime value R’, from 0 to 3, can also be determined from the stress ratio (R) and considering the nature of the vertical stress axes^46,47^. Therefore, the stress regime determines the nature of the vertical stress axes. The stress regime is extensional when σ_1_ is vertical, compressional when σ_3_ is vertical, and strike-slip when σ_2_ is vertical. The stress regime also varies between these three main types; Radial extension (σ_1_ vertical and 0 < R < 0.25), Pure extension (σ_1_ vertical and 0.25 < R < 0.75), Transtensional (σ_2_ vertical and 0.75 < R < 1 or σ_3_ vertical and 0.75 < R < 1), Pure strike-slip (σ_2_ vertical and 0.75 < R < 0.25), Transpression (σ_2_ vertical and 0 < R < 0.25 or σ_3_ vertical and 0 < R < 0.25), Pure compression (σ_3_ vertical and 0.25 < R < 0.75), and Radial compression (σ_3_ vertical and 0.75 < R < 1)^46^. In this study, the Win_Tensor (version 5.9.2) program was explicitly used for paleostress inversion^47^. The heterogeneous data sets were first subdivided into homogeneous subsets and then the reduced stress tensor was calculated from the homogeneous subsets using the PBT method^48,49^ in the Win_Tensor program^47^.

### Direct current (DC) resistivity survey

Subsurface electrical resistivity distribution gives a clear picture of the underlying fractures due to their lower resistivity values in the crystalline hard rock terranes. The subsurface resistance is obtained by passing the electric current through a pair of electrodes and measuring the potential difference through another pair of electrodes^17^. Further, we compute the apparent resistivity information of the subsurface based on the geometrical position of current and potential electrodes. This study carried out the survey using the ABEM Terrameter LS and IRIS SYSCAL Pro instrument.

The electrical data were acquired using two phases during December 2016 and 2018. In the first phase, we collected the data (P2, P3, P7, P11, P12, P13, P14) using the Schlumberger array configuration. Due to the cable length and instrument limitations, we were able to spread the maximum of the profile 235 m with 5 m electrode spacing. Next, we acquired the second phase electrical data (Profiles P1, P4, P5, P6, P8, P9, P10) using the 800 m profile length with 10 m electrode spacing (Table 1). We have collected it using the gradient array configuration. There are two advantages of gradient array over the Schlumberger array measurements. First, the depth of investigation and signal-to-noise ratio is better than the Schlumberger array configuration. Second, the data collection is also rapid in the field. Further, we processed all the measured data before performing the inversion (see Appendix 1).

**Table 1:** Electrical resistivity survey locations in the study area.

ERT profile no	Latitude/Longitude		Elevation range (m)	Location	profile orientation	Zone	Profile length	ERT Configuration
	Initial point	End point						
Profile-1	24.301 72.614	24.304 72.607	288–301	Surela	SE–NW	I	800	Gradient
Profile-2	24.305 72.605	24.304 72.607	301–302	Surela	NW–SE	I	235	Schlumberger
Profile-3	24.297 72.580	24.296 72.582	258–259	Ajapur Mota	NW–SE	I	235	Schlumberger
Profile-4	24.289 72.741	24.291 72.748	425–417	Kengora	WSW–ENE	II	800	Gradient
Profile-5	24.288 72.578	24.294 72.582	272–262	Ghoda	N–S	II	800	Gradient
Profile-6	24.296 72.725	24.289 72.726	396–392	Ajapur Mota	SSW-NNE	II	800	Gradient
Profile-7	24.290 72.576	24.290 72.578	264–274	Ajapur Mota	WSW–ENE	II	235	Schlumberger
Profile-8	24.305 72.675	24.312 72.675	322–321	Padni	SSW–NNE	III	800	Gradient
Profile-9	24.306 72.658	24.299 72.656	307–315	Nichli Ghoda	NE–SW	III	800	Gradient
Profile-10	24.270 72.675	24.274 72.682	318–324	Kanpura	SW–NE	III	800	Gradient
Profile-11	24.295 72.707	24.294 72.709	334–337	Ganji	WNW–ESE	III	235	Schlumberger
Profile-12	24.304 72.589	24.302 72.590	280–283	Kansara	NNW-SSE	IV	235	Schlumberger
Profile-13	24.305 72.602	24.306 72.600	302–301	Surela	NW-SE	IV	235	Schlumberger
Profile-14	24.310 72.675	24.308 72.674	316–312	Padni	NE–SW	IV	235	Schlumberger

#### 2D DC Resistivity imaging

Both forward and inverse modeling has been performed on unstructured meshes to deal with complex subsurface earth structures. Unstructured meshes result in feasible and practical imaging inversion of DC data to deal with a large number of grids^50^. Also, Özyıldırım et al.^51^ compared the rectangular grid-based inversion approach with unstructured meshes. They have concluded that geological boundaries are much cleared in unstructured grid-based inversion as compared to the regular grid. However, the computation of the regularization matrix is much more difficult than regular grid-based.

In the present work, the size of measured field datasets is relatively less than the model parameters i.e., the number of triangles. Thus the optimization problem is extremely underdetermined. We have minimized it via the Gauss–Newton method with regularization by imposing additional model functional as follows^52^:

2 $$\mathop {\min }\limits_{m} \;\Phi^{inv} = \left\| {{\varvec{D}}\left( {\user2{F(m)} - {\varvec{d}}^{{{\varvec{obs}}}} } \right)} \right\|_{2}^{2} + \lambda^{1} \left| {\left| {{\varvec{Rm}}} \right|} \right|_{2}^{2} ,$$

where, $\Phi$ is the objective function to be minimized and $\left\| {} \right\|_{2}^{2}$ is the $\ell^{2}$ norm. $F({\text{m}}) = d^{{{\text{cal}}}}$ is the simulated response and has been computed by solving related partial differential equations by following the procedure of Rucker^53^. $D$ is called the data weighting matrix and it is related to *i*th observation of apparent resistivity data^54^. $\lambda^{1}$ is known as the regularization parameter and controls the compromise between data error and model function. In Eq. (2), regularization matrix *R* is the Laplacian operator i.e., second-order differential operator. The computation of the second-order differential operator for rectangular grid discretization is well known for a decade. However, it is relatively new and challenging as there is very little literature available^50^. During the iterative minimization of Eq. (2), the following root means square values (Eq. 3) were computed at every inversion step^55^.

3 $$RMS= \sqrt{\frac{({D}\left({d}^{obs}-{d}^{cal}\right){)}^{T} ({D}\left({d}^{obs}-{d}^{cal}\right))}{N}}$$

where *N* is the total measured data sets. The inversion will stop if the root means square error will no longer be reduced. In the present work, we have modified the inversion approach of Singh et al.^56^ to the unstructured meshes. Further, Appendix 1 shows the summary of forward/inversion parameters.

#### Model uncertainty analysis

As far as uncertainty is concerned, the model resolution matrix was calculated to estimate the resolution quality of final resistivity models obtained from apparent resistivity data. We have computed the model resolution matrix **R**^**m**^ using the Eq. (4)^57^:

4 $${{\varvec{R}}}^{{\varvec{m}}}=({{{\varvec{J}}}^{{\varvec{T}}}{{\varvec{D}}}^{{\varvec{T}}}{\varvec{D}}{\varvec{J}}+{{\varvec{\uplambda}}}^{1}{{\varvec{R}}}^{{\varvec{T}}}{\varvec{R}})}^{-1}{{\varvec{J}}}^{{\varvec{T}}}{{\varvec{D}}}^{{\varvec{T}}}{\varvec{D}}{\varvec{J}}$$

where ***J*** is a matrix of partial derivatives obtained after the final iteration of the inversion. The diagonal elements **R**^**m**^ are displayed as 2D model section and interpreted with the corresponding inversion result.

**Figure 2:**
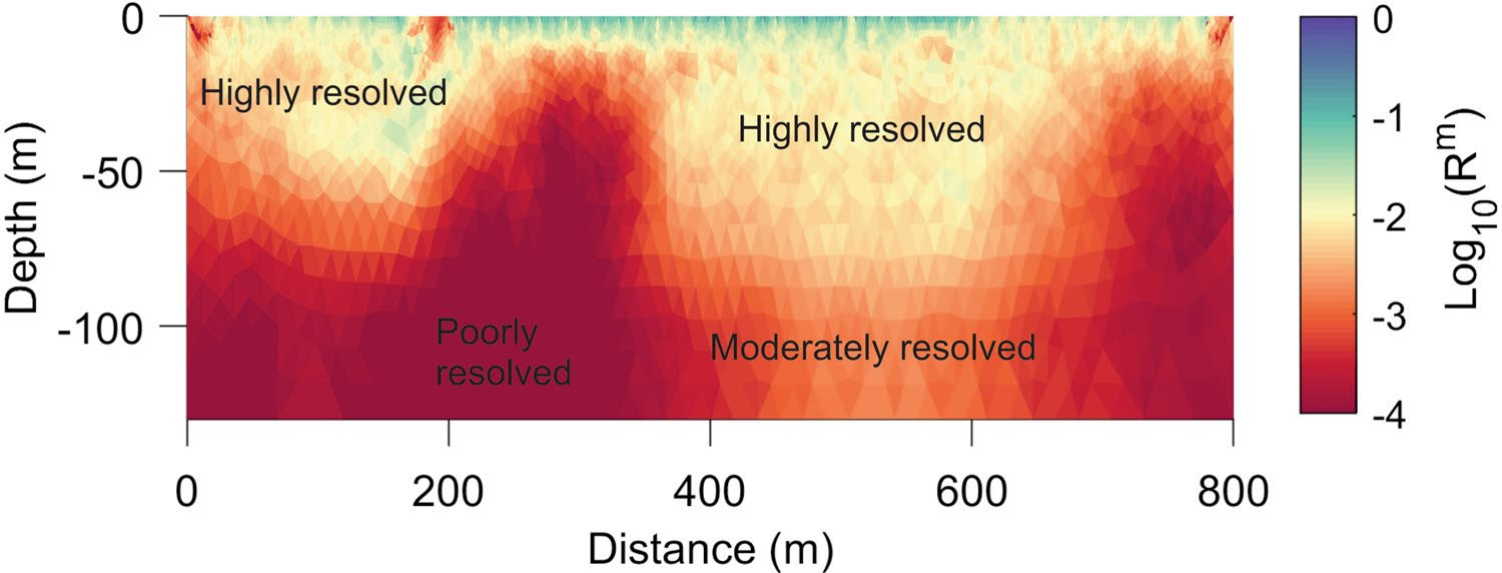
Diagonal elements of the model resolution matrix for the inverted resistivity model (Profile-4).

The model resolution explains which part of the resistivity model is solved sensitively. Figure 2 shows the diagonal elements of the model resolution matrix for the inverted resistivity model for Profile-4 (refer to “Zone-III: Localities with fracture and faults (Brittle zones)”, Fig. 14a). Theoretically, the best solution is obtained when the diagonal elements of the model resolution matrix are equal to one, and the rest of the component in the same row is zero^58^. However, in practice, it is normally observed that these diagonal element values are less than one in the 2D inversion of DC resistivity data^58^. Özyıldırım et al.^51^ showed that when the common (base-10) logarithm of a resolution value (i.e., log10 R^m^) is greater than − 2.5, the related model parameter is very well resolved. Further, it can be clearly seen in Fig. 2, that the majority of the logarithm of resolution values are very high (green to light yellow zones). Thus the delineated subsurface resistivity model at Profile-4 has high sensitivity. However, the dark-colored zone represents the logarithm resolution values as less than -3.0. In this zone, the data density is sparse and thus is not well resolved. We have performed this test for all acquired resistivity profiles in the study region.

## Results and interpretation

### Structural analysis

**Figure 3:**
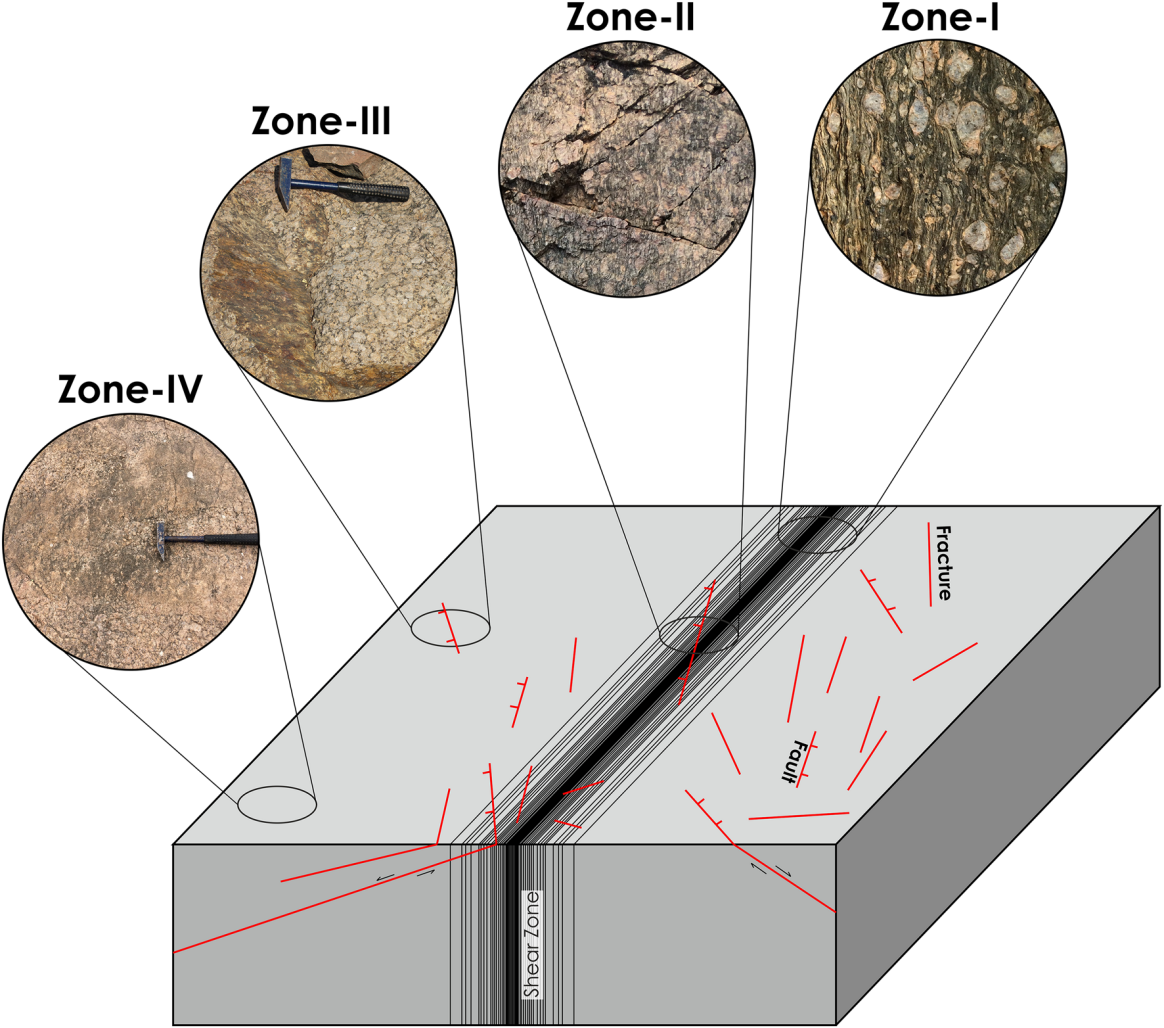
Sketch showing the different zones and corresponding structural features. Zone-I, the domain with pervasive foliation fabrics. Zone-II, the domain with both ductile foliation fabrics and brittle faults and fractures. Zone-III, domain with brittle faults and fractures only. Zone-IV, the domain devoid of observable ductile (foliations) and brittle structures (fractures and faults).

Based on the presence of brittle faults, fractures, and ductile shear zones in the field, the study area is subdivided into four different domains (Fig. 3).

1. Domain with ductile shear zones only (Zone-I): In a few localities, the pervasive mylonitic foliations in ductile shear zones missed the brittle faulting and fracturing as observed in outcrops. The ductile foliation fabric is the dominant planar structure in these zones.
2. Domain with ductile shear zones overprinted by late brittle faults and fractures (Zone-II): Shear zones were predominantly overprinted by brittle fractures and faults.
3. Domain with brittle faults and fractures only (Zone-III): These domains are devoid of pervasive foliations but over-printed by brittle fractures and faults.
4. Domain devoid of fractures, faults, and shear zones (Zone-IV): These domains contain no observable pervasive foliations and are also devoid of brittle deformation structures such as fractures and faults.

#### Ductile shear zones

**Figure 4:**
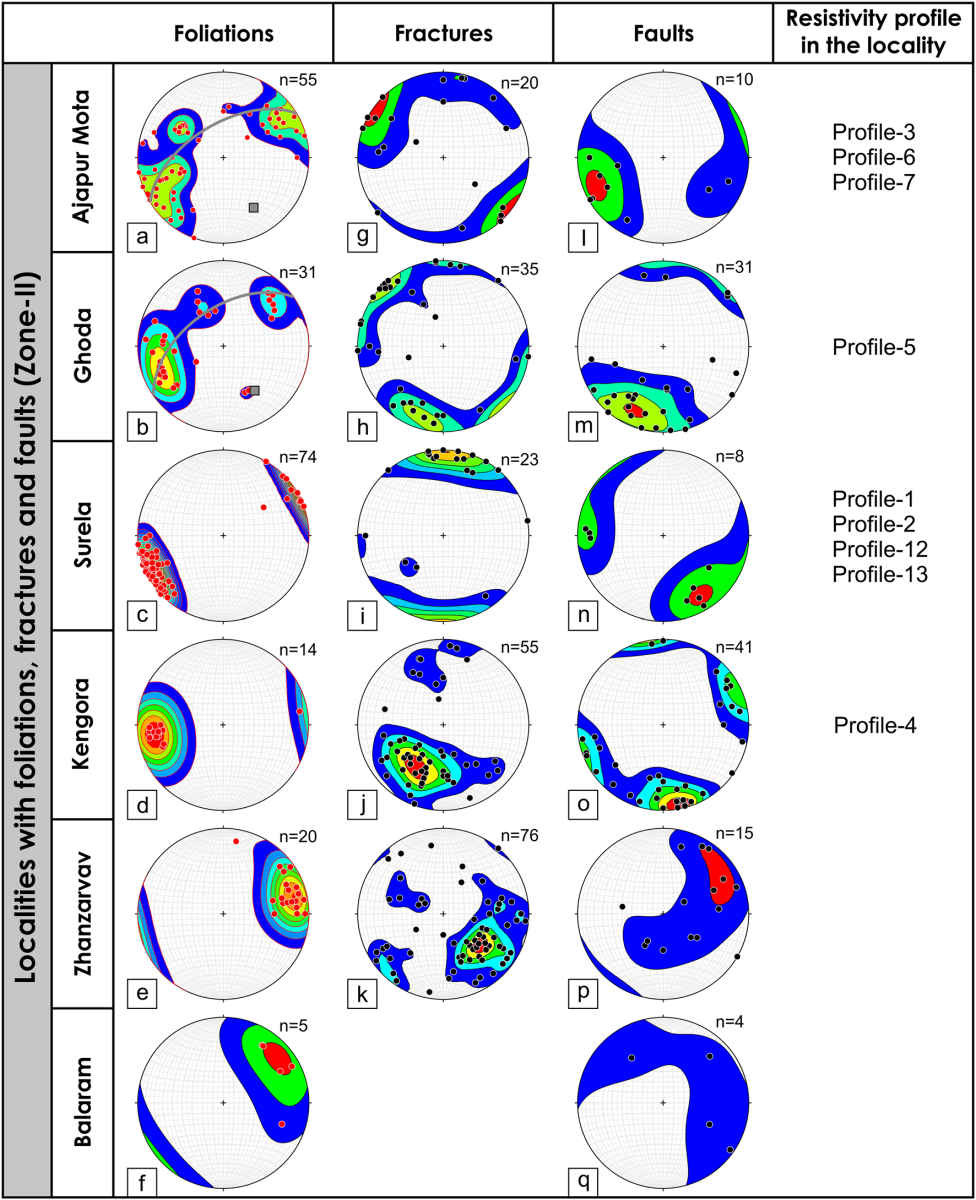
Poles to the foliations, fractures, and faults for different localities from Zone-II are plotted in the lower hemisphere stereographic projection along with the resistivity profile number in the locality. (**a**–**f**) Poles to the foliations from different localities are plotted in lower hemisphere projection. The poles to the foliations from localities, Ajapura Mota and Ghoda represent a girdle distribution, indicating folding of foliations. The gray square represents the poles to the best-fit girdle is the fold axis, (**g**–**k**) Lower hemisphere projection of poles to the fractures from different localities, (**l**–**q**) Lower hemisphere projection of poles to the fault surface collected from different localities.

**Figure 5:**
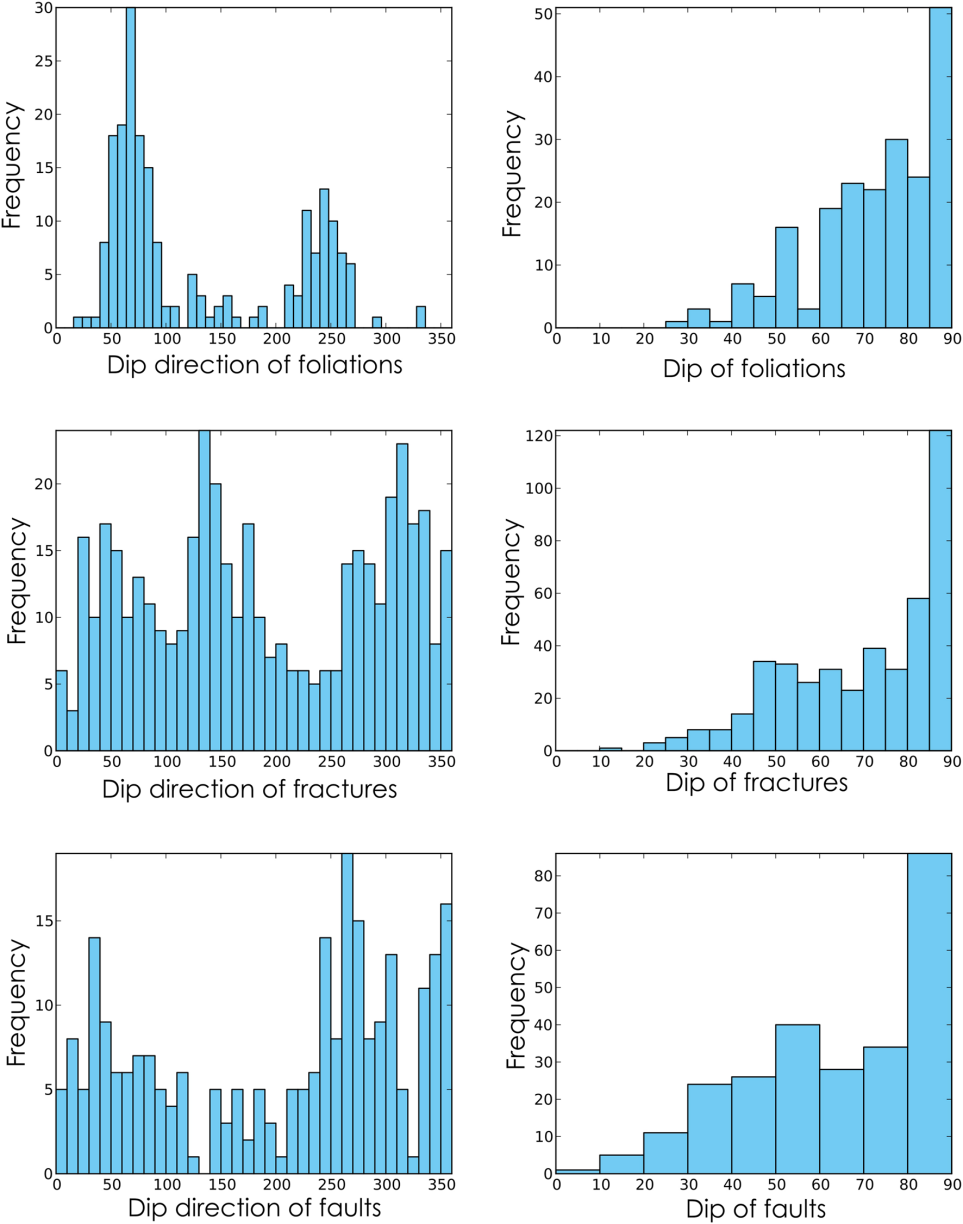
(**a**,**b**) Histograms plotted for dip direction and the dip amount of foliations, (**c**,**d**) Histograms plotted for dip direction and dip of fractures, (**e**,**f**) Histograms plotted for dip direction and dip of faults.

Several NW–SE trending vertical to sub-vertical shear zones dissect the study area as shown in the Fig. 1. The foliation fabrics and shear sense indicators strongly suggest that these shear zones are mostly sub-vertical shear zones with a thrust to strike-slip type shear sense. Some of the shear zones change the trend from NW–SE to ENE–WSW (SZ-II) and NE–SW (SZ-I, SZ-IV, and SZ-V) (Fig. 1). The shear zones not only change their strike but also change the dip along the strike (SZ-I, SZ-IV, and SZ-V). The lower hemisphere projection of the foliation data depicts that the foliations are mostly steeply dipping along NE-SW (Fig. 4). In two localities, Ajapur Mota and Ghoda, the foliations are folded inferred from the girdle distribution of the poles to the foliation surfaces (Fig. 4). This is likely because of the change in trend and dip of the shear zones in these localities, observable in the geological map (Fig. 1). The foliations are dominantly NE-SW trending. However, three orientation clusters are distinguishable, NE-, SE-, and SW dipping foliations (Figs. 4 and 5a). Foliations are dominantly vertical with an average dip of 71° (Figs. 1 and 5b).

**Figure 6:**
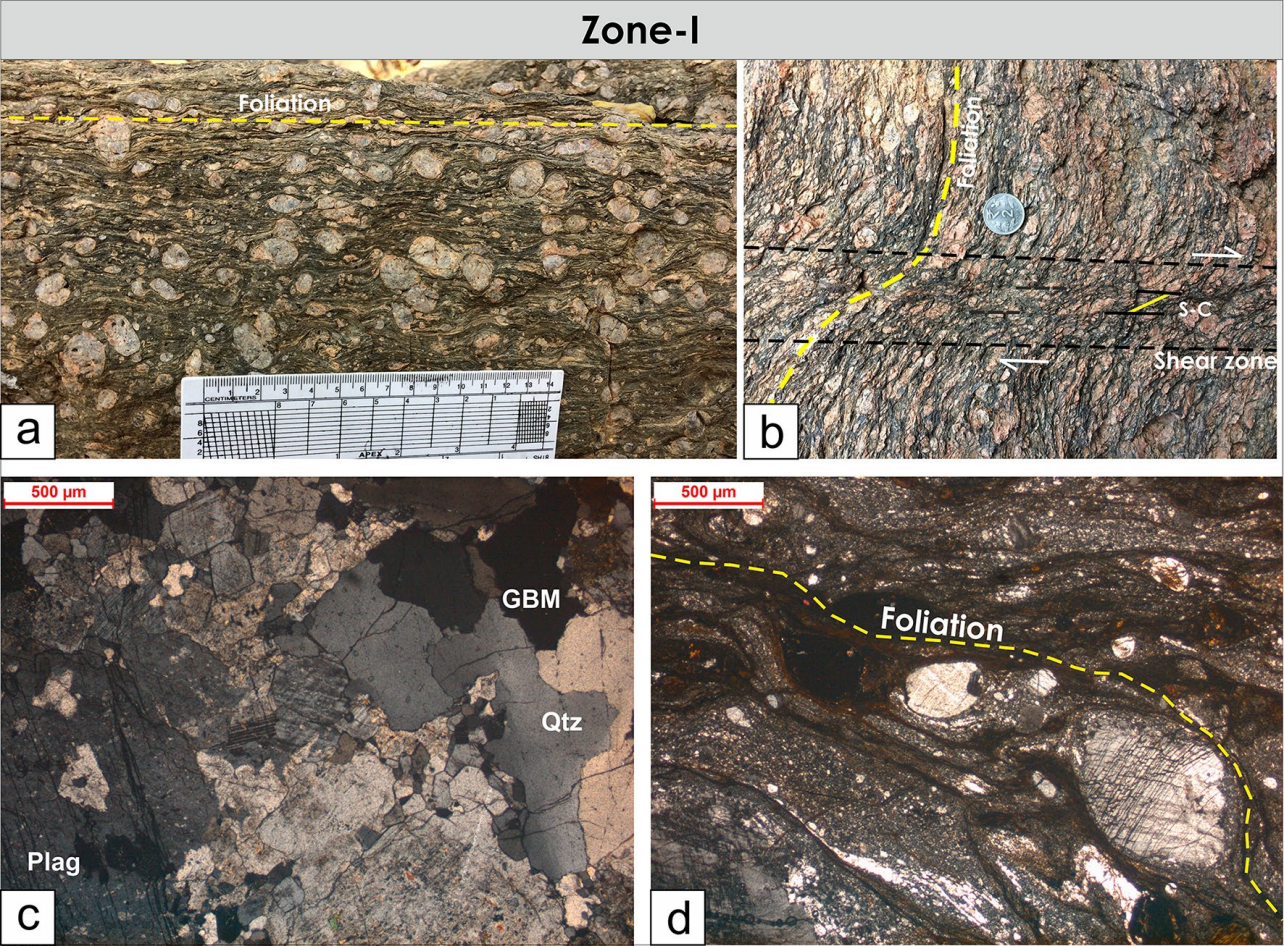
Field photographs and photomicrographs of structures from Zone-I. (**a**) Granite mylonite with mylonitic foliations and rotated feldspar porphyroclasts, (**b**) A localized shear zone with S-C fabrics crosscut the mylonitic foliations, (**c**) Photomicrograph of the granite mylonite with crystal plastic deformation (Grain Boundary Migration, GBM) in quartz and feldspar, (**d**) Photomicrograph of mylonite with typical core-mantle structures in feldspar.

Both in the outcrop scale and under the microscope, the mylonitic texture is dominant in Zone-I with deformed and rotated feldspar porphyroclasts (Fig. 6). The crystalloplastic deformation structures (Subgrain rotation to grain boundary migration) in both quartz and feldspar can be seen in thin sections (Fig. 6). Considerable grain size reductions in the shear zone are because of intense shearing observable in the thin section (Fig. 6). In outcrops and under the microscope, the mylonites can be vaguely classified into proto-mylonite (modal percentage of matrix grains less than 50%) to meso-mylonite (modal percentage of matrix grains between 50 and 90%) types. Typical core-mantle structure in feldspar can also be seen under the microscope (Fig. 6). All the deformational microstructures depict a higher temperature of deformation between 500 and 700 °C^57^.

#### Brittle fractures and faults

**Figure 7:**
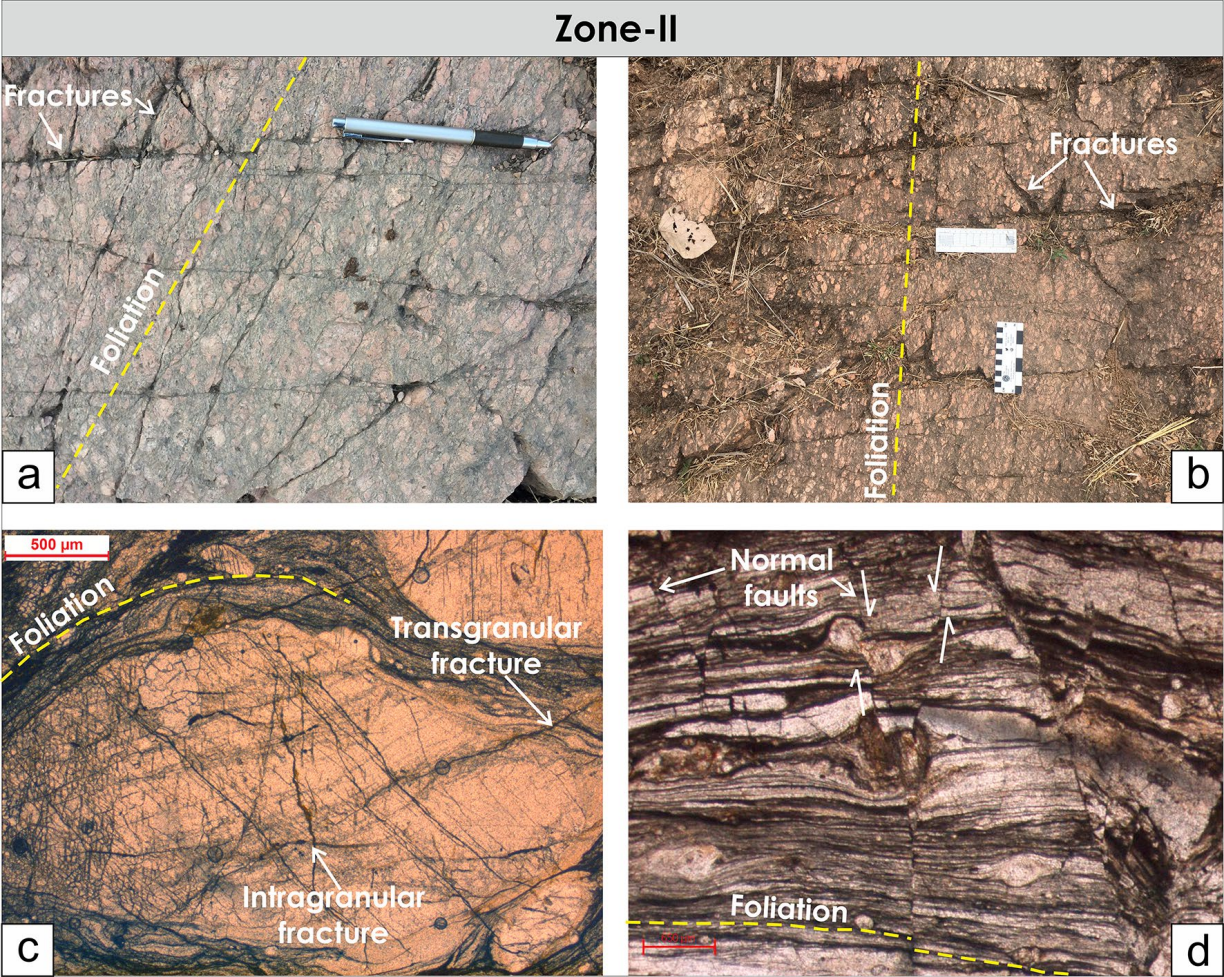
Field photographs and photomicrographs of rocks and structures from Zone-II. (**a**,**b**) Fractures overprinting the pervasive foliations in the mylonites, (**c**) Photomicrograph of mylonite with typical core-mantle structure in feldspar with both intragranular and transgranular fractures, (**d**) Photomicrograph of mylonite overprinted by brittle faults with a dominant normal sense of shearing.

**Figure 8:**
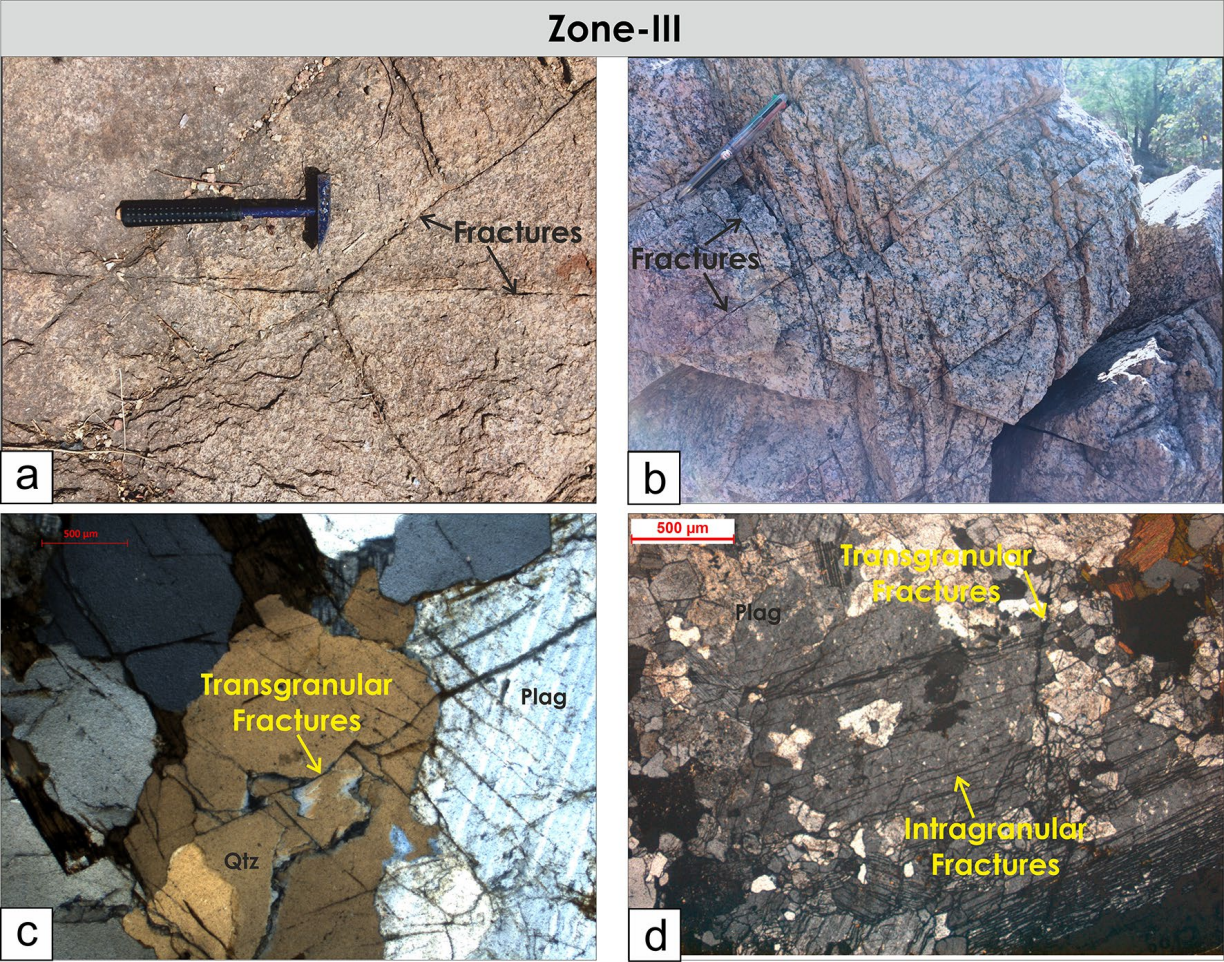
Field photographs and photomicrographs of rocks and structures from Zone-III. (**a,b**) Brittle fractures and faults overprint the massive granites. (**c,d**) Both intragranular and transgranular microfractures observed under the microscope.

**Figure 9:**
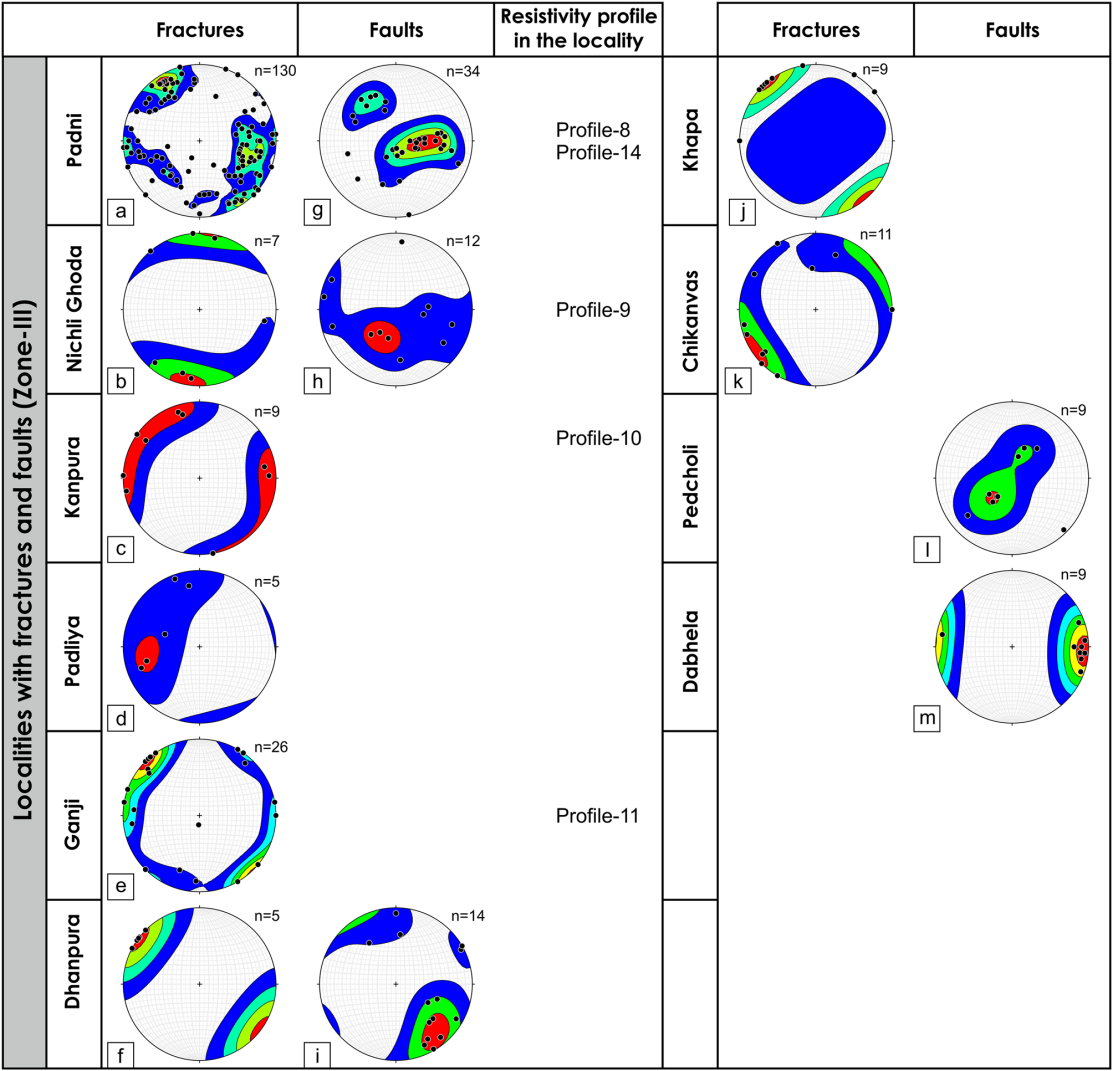
Poles to the fractures and faults for different localities from Zone-III are plotted in the lower hemisphere stereographic projection along with the resistivity profile number in the locality. (**a**–**f,j,k**) Lower hemisphere projection of poles to the fractures from different localities, (**g–i,l,m**) Lower hemisphere projection of poles to the fault surface collected from different localities.

**Figure 10:**
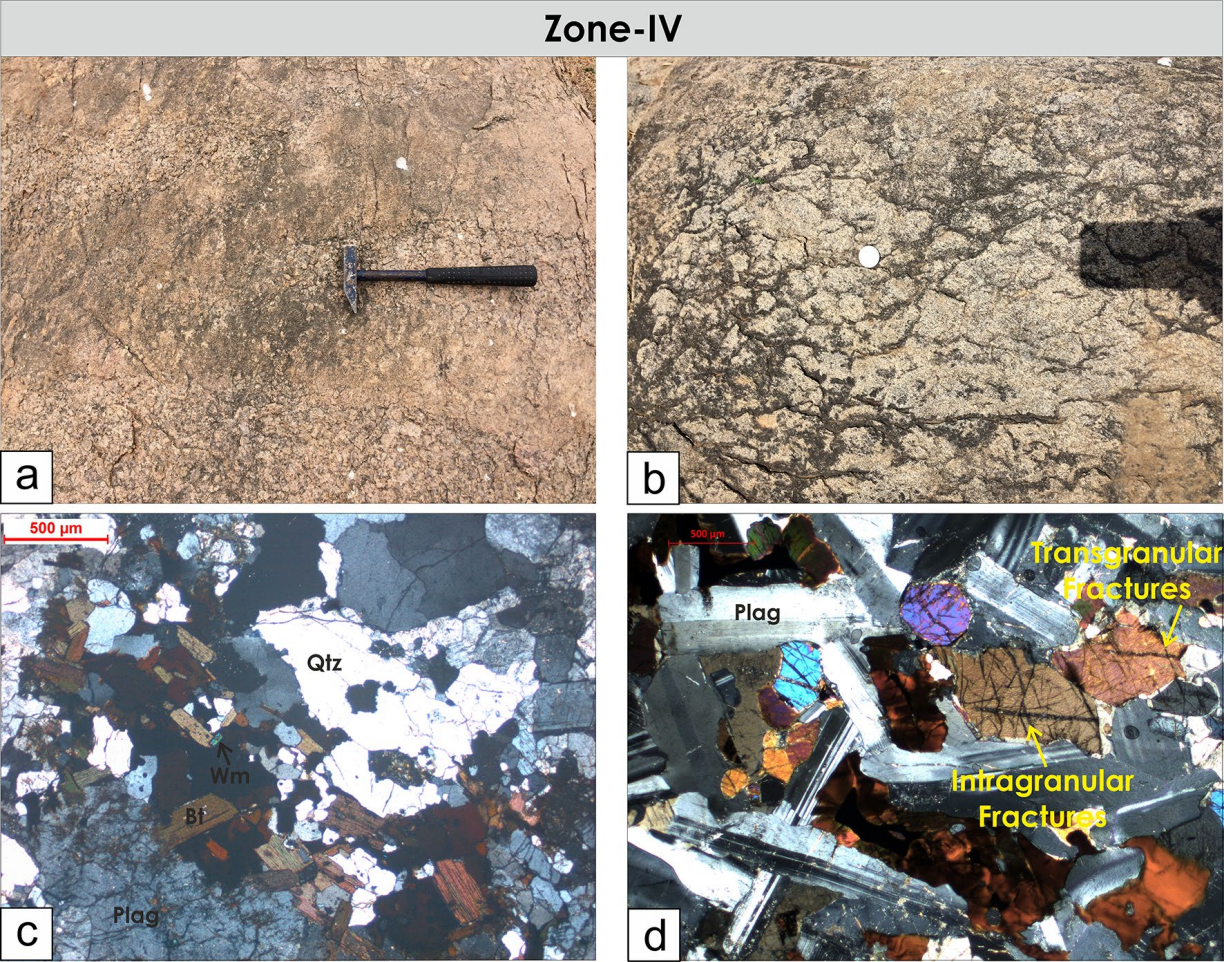
Field photographs and photomicrographs of rocks from Zone-IV. (**a,b**) Lack of observable fractures and faults in outcrop scale. (**c,d**) Seldom occurrence of both inter-and transgranular fractures under the microscope.

The cross-cutting relationships between the foliations and brittle fractures and faults can be seen in Zone-II and Zone-III from the outcrops and also from microscopic observations where the late brittle fractures and faults cross-cut the mylonitic foliations (Figs. 7, 8). In many localities, a good correlation between the fracture and faults is observed (Figs. 4, 9). The fractures are mostly moderate to steeply dipping with a strong variability in dip direction (Figs. 4, 5, 9). However, three distinct clusters in the dip directions of fractures can be seen i.e., NE-, SE-, and NW dipping (Figs. 5, 9). The fault data also demonstrate three distinct clusters in the dip direction i.e., NE-, E-, and NNW dipping (Fig. 5a). In thin sections, both intra-crystalline and inter-crystalline fractures were observed (Figs. 7, 8). These above-mentioned observations suggest that a late brittle deformation (faults and fractures) overprints the shear fabrics. In the field, a few ductile shear zones are devoid of late brittle faults and fractures, and a majority of them are overprinted (Fig. 6). In some localities, massive rocks, devoid of ductile shearing and brittle fracturing in both outcrop scale and under the microscope can be observed (Fig. 10).

**Figure 11:**
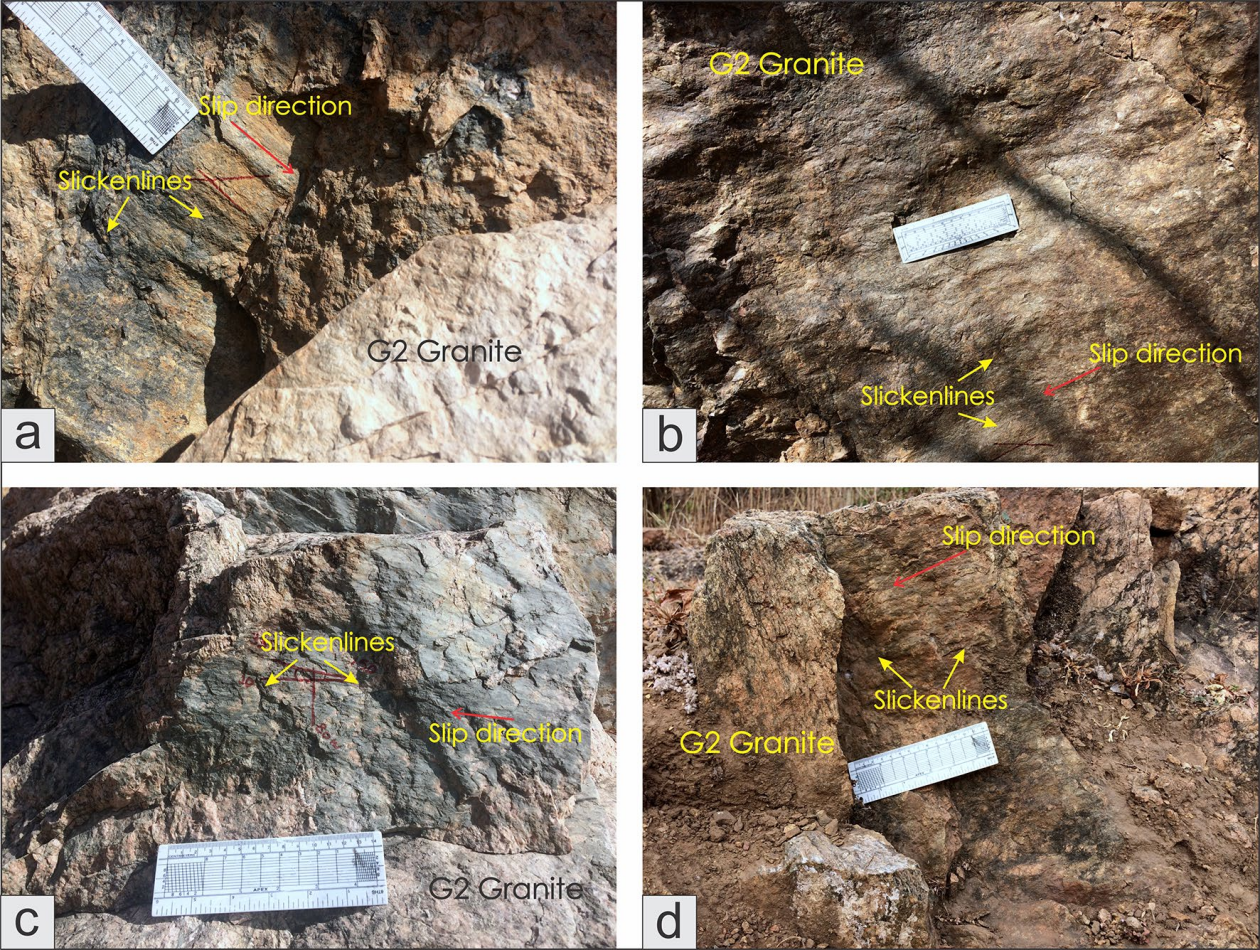
Observed fault surfaces with slicken fibers in the field. (**a**) Moderately dipping fault surface with normal slip, (**b**) A gently dipping oblique-slip fault, (**c**) Steeply dipping strike-slip fault with a sinistral sense of shearing, (**d**) A moderately dipping oblique-slip fault.

**Figure 12:**
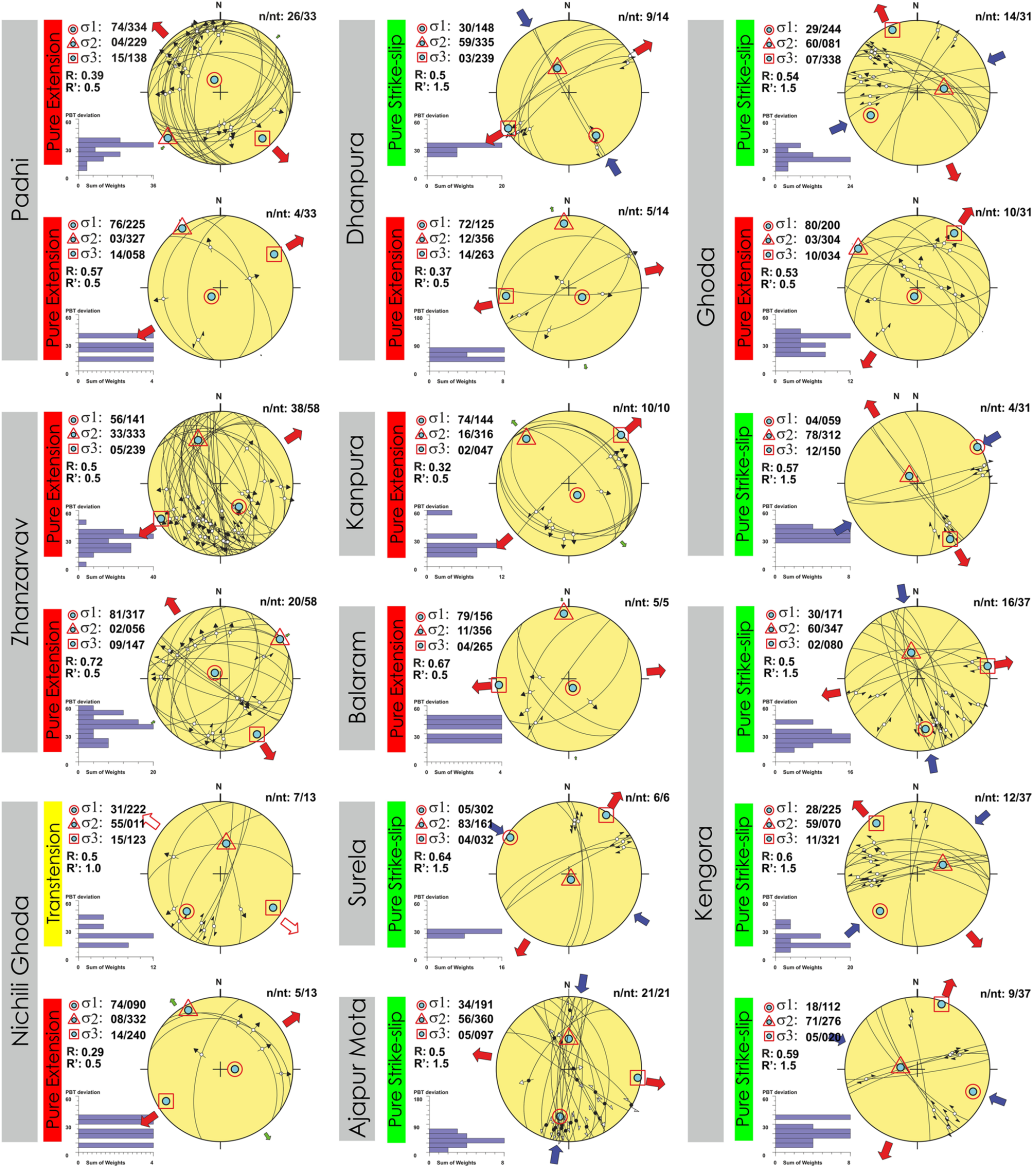
Paleostress analysis of fault-slip observations from different localities using the PBT method^48,49^. At different localities, the heterogeneous fault slip-observations were classified into different homogeneous subsets. *n* Number of data in a homogeneous subset. *nt* Total number of data measured. The histogram represents PBT deviations. Angular deviation approaches zero for best-fit data. R (Stress ratio) = (σ2 − σ3/σ1 − σ3). R′ = Stress regime. Pure strike-slip and pure extension regimes predominate the area.

A total of 228 fault slip data were analyzed from the study area for paleostress estimations. The mesoscopic fault surfaces contain quartz and/or calcite slicken fibers (Fig. 11). The fault-slip data collected from Zone-II and Zone-III were arranged based on their localities and subdivided into homogeneous subgroups for paleostress estimations. Paleostress estimations indicate invariant NW–SE to NE-SW extensions in all the localities. No considerable fluctuation in the stress regime can be observed with two distinct extension events i.e., NE-SW extension and NW–SE extension (Fig. 12). All the faults are formed in a pure extension and strike-slip stress regime except in some of the faults in the Nichli Ghoda region formed in a transtension regime (Fig. 12). The stress ratio fluctuates between 0.29 and 0.72 (Fig. 12).

### Geophysical investigations

The depth of bedrock and basement topography is vital to understand the subsurface geological formations. Based on the observations from the shear zones, fractures, and faults in the study area, fourteen electrical resistivity profiles were taken for detailed analysis for the four different zones i.e., Zone-I, Zone-II, Zone-III, and Zone-IV (Table 1).

#### Zone-I: Localities with foliations (Ductile shear zones)

**Figure 13:**
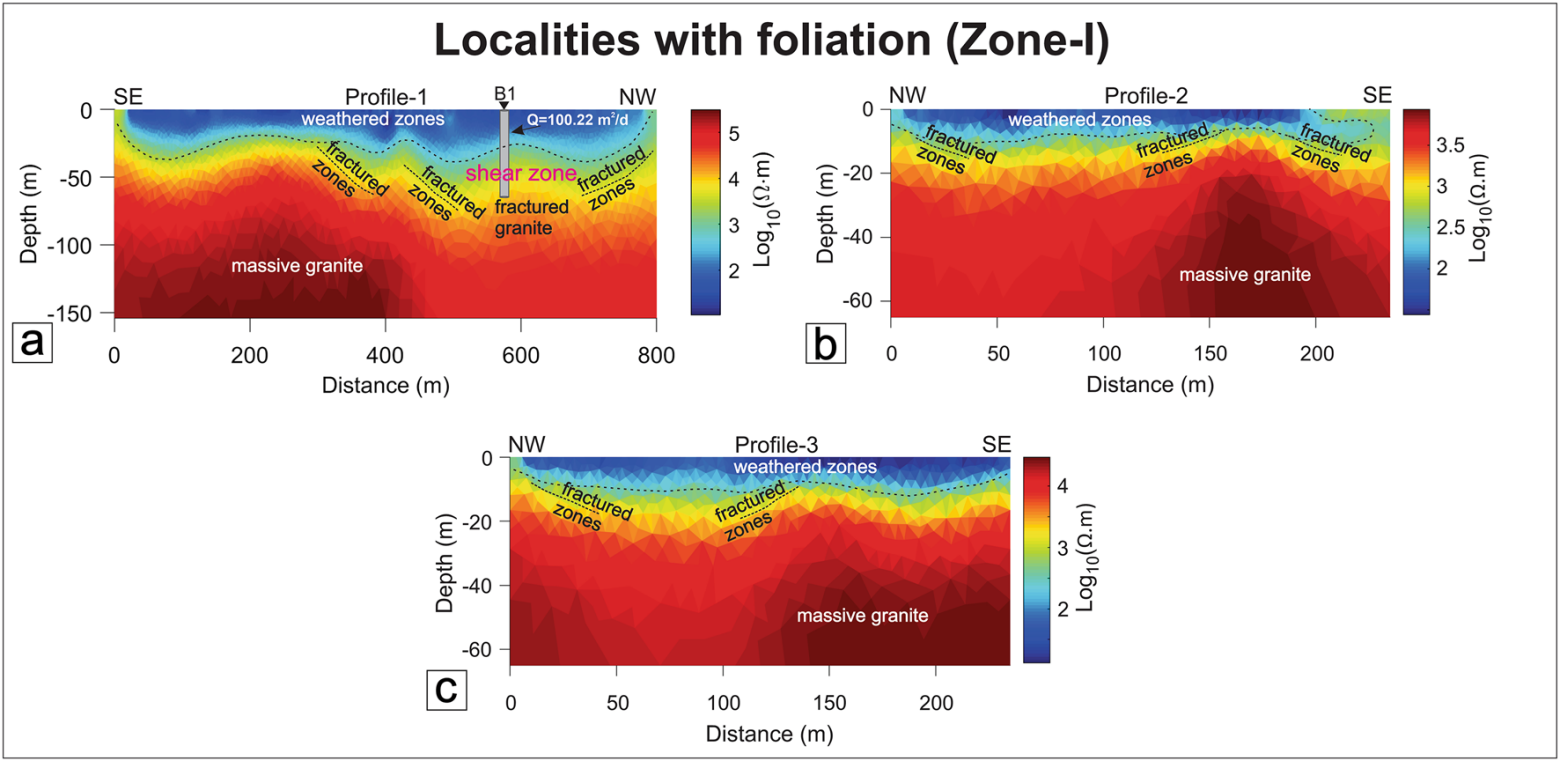
Resistivity profiles in Zone-I. (**a**) Profile-1, Surela area, (**b**) Profile-2, Surela area, (**c**) Profile-3, Ajapur Mota area. B1 shows the borehole location and the discharge rate (Q) in the Surela area.

Three profiles (P1–P2: Surela and P3: Ajapur Mota) have been performed parallel/subparallel to the shear zones (Fig. 1). P1 and P2 traverses have performed subparallel to the Surela shear zone (SZ-III) along the NW–SE direction (Fig. 13a,b). The rock type of the study area is predominantly G2 granite which was mylonitized due to strike-slip shearing (Fig. 13a,b). The interpreted resistivity model exhibit values ranging from 10 to about 10^5^ Ω-m with two distinct features i.e., minor weathered-fractured zones and massive granite as the basement. The weathered thickness in this area varies from 1 to 20 m with a maximum depth of 50 m with a few sets of shallow fractures dipping towards NW and SE, observed in the resistivity section. P3 traverse is taken in the northeastern flank of the Ajapur Mota shear zone (SZ-IV) (Fig. 13c) which shows similar features as P1 and P2 with an absence of any fractures in the resistivity section. In these localities, no such deep-seated weathered zones were found where water can be stored, although pervasive foliations are well present. Further, these subsurface features are in good agreement with the geological map.

#### Zone-II: Localities with foliation, fractures, and faults (Brittle-Ductile zones)

Four ERT traverses (P4: Kengora, P5: Ghoda, and P6–P7: Ajapur Mota) have been taken along/across the major shear zone areas with major/minor faults and fractures.

**Figure 14:**
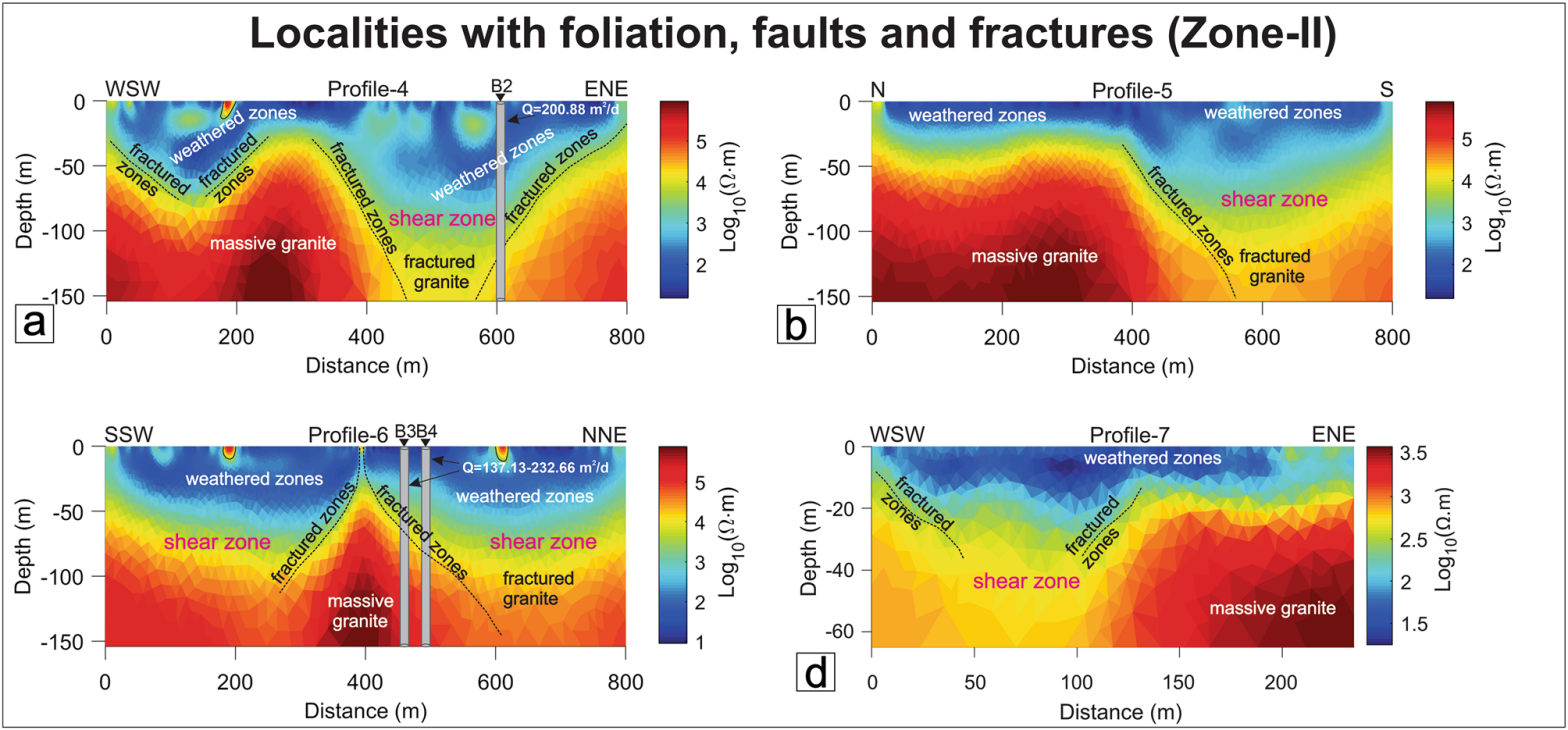
Resistivity profiles in Zone-II. (**a**) Profile-4, Kengora area, (**b**) Profile-5, Ghoda area, (**c**) Profile-6, Ajapur Mota area, (**d**) Profile-7, Ajapur Mota area. B2, B3, and B4 represent the borehole locations and their respective discharge rates (Q) in the Kengora and Ajapur Mota areas.

Profile-4 was collected using the gradient configuration in Kengora shear zone (KSZ) (SZ-I) (Fig. 1). The orientation of the resistivity profile is ENE-WSW, orthogonal to the KSZ. In this area, the G2 granites are highly mylonitized. The resistivity model shows three features namely, top weathered zone, deep fractured zone (sub-vertical), and massive granite/mafic granulite body (Fig. 14a). The resistivity model depicts a vertical to sub-vertical boundary of resistive and conductive zones at 400 m profile location. The boundaries show sub-vertical and in good pact with the regional geological map (Fig. 1), where a shear zone is likely in this position. The resistivity value varies from less than 10 to 10^5^ Ω-m. It depicts that the distinct top layer is highly weathered zones with an average thickness of 50 m. Further, it comprises weathered rock, clay, and silt with a resistive value ranging from 30 to 500 Ω-m. The weathered granite extends to the partly fractured granite with ~ 150 m thick and its resistivity varying from 500 to 5000 Ω-m. The low resistivity zone indicates the most promising water-bearing zones that are capable of groundwater storage.

Profile-5 (Figs. 1, 14b) has been performed using a similar configuration as P4 which is sub-parallel to the N-S trend Ghoda shear zone (GSZ). A WNW-ESE trending major fault (~ 15 km) cross-cut the GSZ in the southern end. The GSZ is one of the major shear zones that initially trend in N-S orientation and later bent down towards the E-W. The GSZ comprises mainly G1 granite. The resistivity value in this area varies from 10 to 10^5^ Ω-m similar to P4. The profile shows a weathered zone consisting of clay and soil, a deep fractured zone towards the south, and massive granite/mafic granulite towards the northern part. The weathered zones show a thickness ranging from 40 to 50 m followed by a few sub-vertical fractured zones dipping towards the south. Although the area accommodates a shear zone which is highly deformed in nature, the northern end of the profile shows high resistive and is mostly massive below the depth of 40–50 m. It may be due to the resistivity traverse being parallel to the shear zone direction and no such fractures/faults were crossed over it. However, a distinct low resistive stripe width of 400 m has been located at 400–800 m in the resistivity section (Fig. 14b). The zone shows a ~ 120 m thick conductive zone and is relatively a suitable reservoir. This is due to a WNW-ESE-oriented fault overprinting the shear zone.

Profile-6 and Profile-7 ERT profiles have been collected across the NW–SE-oriented Ajapur Mota Shear zone (SZ-IV) (Fig. 1). In this area, granite has been highly sheared and mylonitised and a NE-SW fracture has crossed over the shear zone (Fig. 1). The resistivity models show the distinct conductive and resistive zones and the resistivity distribution of top weathered zones, fractured granite, and massive granite as the basement (Fig. 14c,d). The thickness of the weathered zone is ~ 100 m and is almost uniform throughout the profile except in the central area.

#### Zone-III: Localities with fracture and faults (Brittle zones)

**Figure 15:**
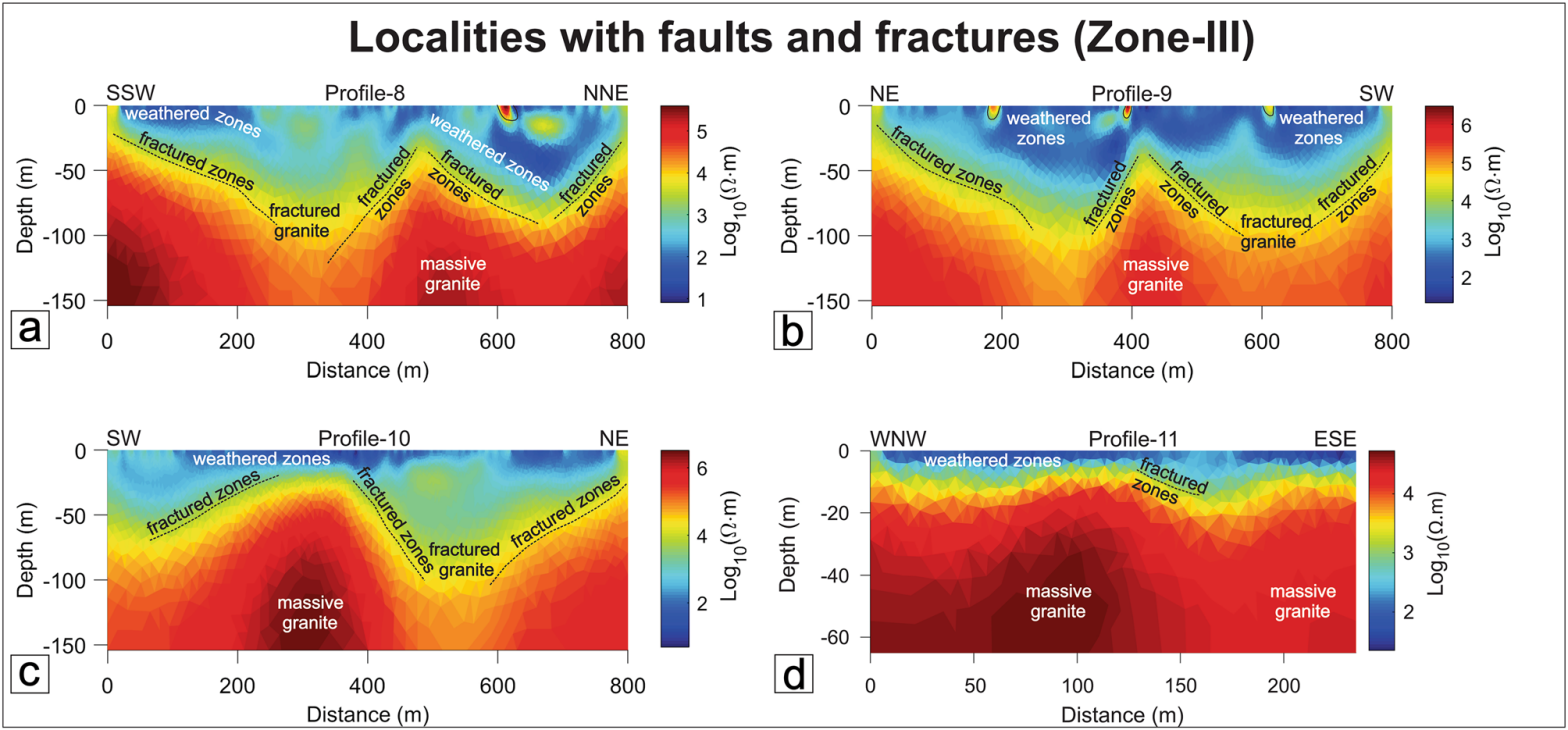
Resistivity profiles in Zone-III. (**a**) Profile-8, Padni area, (**b**) Profile-9, Nichli Ghoda area, (**c**) Profile-10, Kanpura area, (**d**) Profile-11, Ganji area.

Four ERT traverses (P8-P11) have been performed along and across the fault/fracture zones (Figs. 1, 15a–d). The interpreted resistivity model shown in Profile-8 has two different layers, weathered/fractured granite and massive granite/granulite (Fig. 15a). The model depicts that the distinct top layer of P8 is mixed with weathered and fractured granite. Profile distances from 210 to 350 m and 500–700 m, distinct fractured zones have been encountered, which lie just below the subsurface. The fractured zone is formed due to the WNW-ESE trend large-scale fault. Further, the thickness of weathered and fractured granite is nearly 70 m with resistivity values ranging from 10 to 500 Ω-m, as seen in the resistivity depth section. These low resistivity values are the most promising water-bearing zone in the study region. Massive granite (basement rock) is encountered below the fractured/weathered granite layer with an average depth of 50–60 m (Fig. 15a).

Similar to Profile-8, the orientation of Profile-9 is in the NE-SW direction and the interpreted resistivity model shows two major layers namely highly weathered-fractured granite and massive granite/granulite (Fig. 15b). An NNE-SSW trending fracture has been noted closed to the resistivity traverse. The resistivity section shows mixed-up resistivity values i.e., low, moderate, and high resistive zones. The top 10–15 m show very low resistivity ranging from 10 to 100 Ω-m followed by moderate resistivity, ranging from 50 to 3000 Ω-m approximately. The maximum thickness of top layered granite is towards the northern end of the cross-section. A vertical to sub-vertical fracture zone is encountered at 400 m in the resistivity section. The fracture zone demarcates the contact between two lithologies, namely granite and mafic granulite (Fig. 15b). The low resistivity zones in this area suggest the capability to store groundwater. The central part of the section is massive and is not suitable for groundwater storage due to the lack of void/fracture spaces.

Profile-10 is collected along the SW-NE direction. The thickness of the top weathered zone is much less than other profiles collected in the study region. The identified fracture zone has been marked in the resistivity section. The profile shows the second layer (fractured zone) variation along the profile which is nearly 60 m thick at a profile distance starting from 450 to 600 m. This zone could be a potential site where recharge will be more. Like other profiles, massive granite is present in the basement (Fig. 15c).

Profile-11 is oriented in the WNW-ESE direction. The weathered zone thickness is very less compared to other resistivity profiles in the study region. Small scale fractured zones were identified and marked in the resistivity section (Fig. 15d). Although the ERT profile is taken on a brittle zone, no such deep fractured zone has been identified. The resistivity model shows two distinct resistivity zones. The top 10-20 m show very low resistivity values ranging from 10 to 100 Ω-m followed by massive granite/granulite of 500–3000 Ω-m.

#### Zone-IV: Localities devoid of foliation, fracture, and faults (undeformed zones)

**Figure 16:**
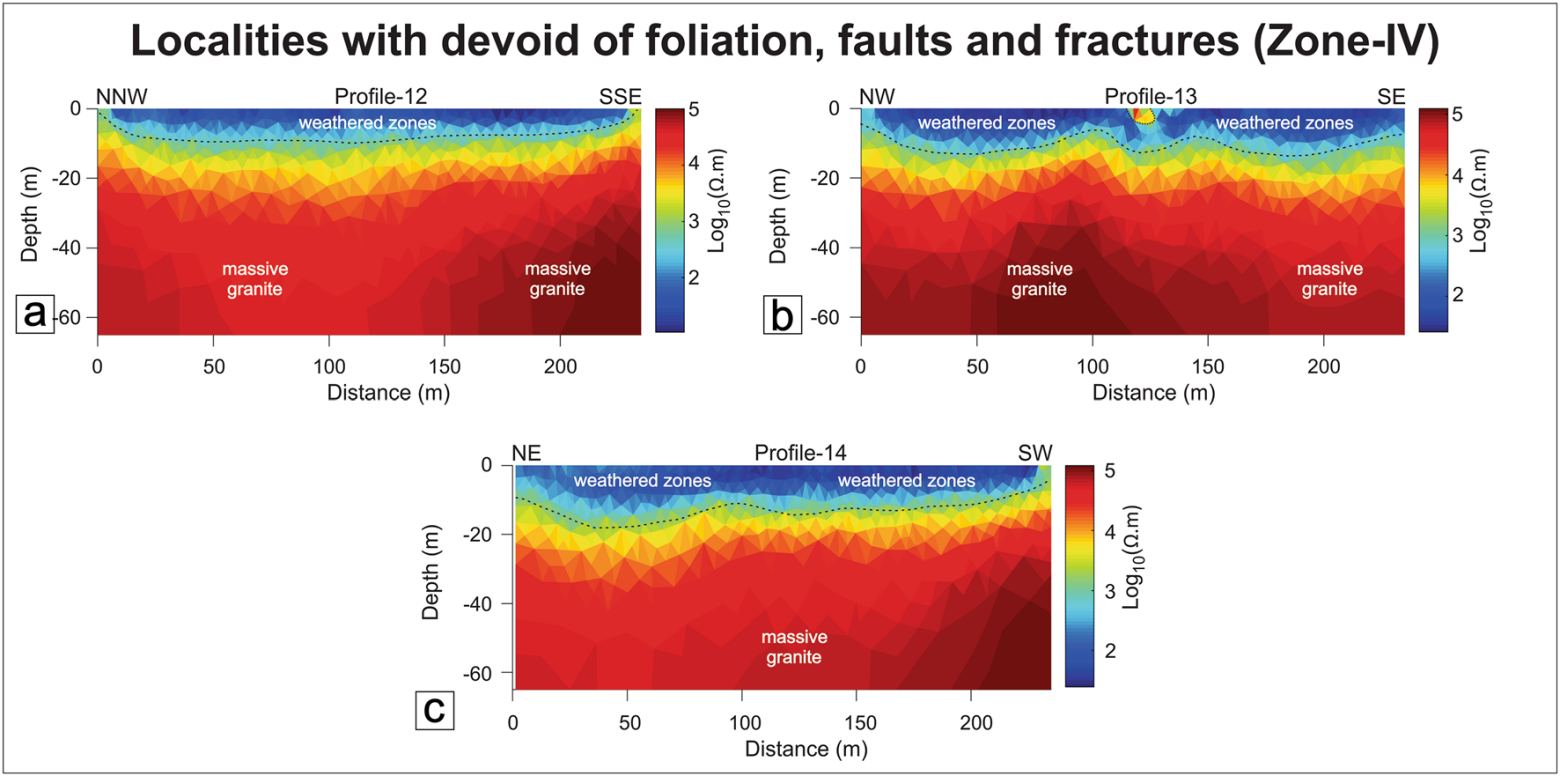
Resistivity profiles in Zone-IV. (**a**) Profile-12, Kansara area, (**b**) Profile-13, Surela area, (**c**) Profile-14, Padni area.

Three ERT traverses (P12:Kansara, P13:Surela, P14:Padni) have been performed over massive granite or mafic granulite to understand the resistive nature of the subsurface (Figs. 1, 16a–c). The observed resistivity model shows low resistivity zones extending up to 15 m. These zones vary across the traverse of each section which shows the weathered profile variability (5–15 m). The resistivity value ranges from 10 to ~ 10^5^ Ω-m with two discrete features such as top weathered zones and highly resistant massive granite/mafic granulite below the weathered zone (Fig. 16a–c).

### Precipitation, groundwater table, and aquifer parameters

**Figure 17:**
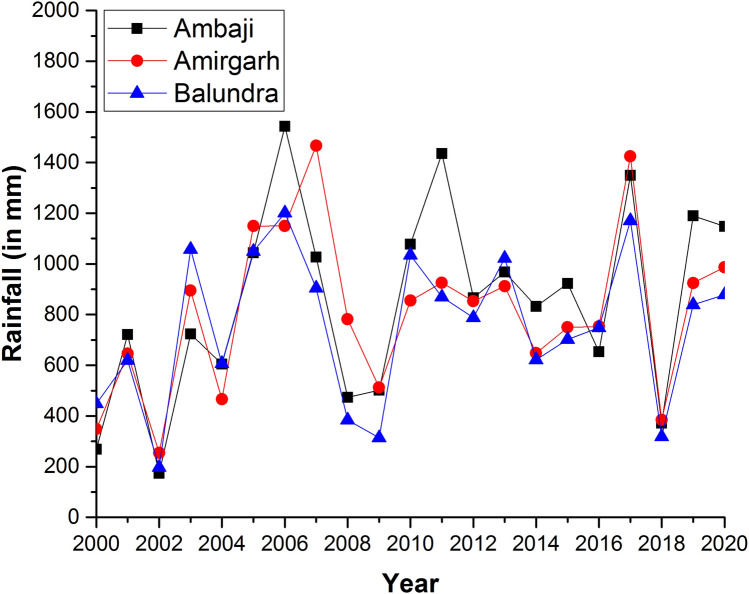
Decadal precipitations (2000–2020) data in the study area compiled from Water Resources Department, Govt. of Gujarat. (Source: https://swhydrology.gujarat.gov.in/).

In this study, three rainfall stations were taken into consideration to understand the precipitation pattern in the last two decades. It shows that in a particular year, the precipitation almost remains the same throughout the region (Fig. 17). The groundwater table depth varies from a meter to several meters in the study region. The water table data displays that the area bounded between the Surpagla-Kengora- and Kui-Chitraseni-fault is relatively low water table depth (see Appendix 2,3) both in pre-monsoon and post-monsoon periods. Further, the aquifer parameter, transmissivity (T) of eight boreholes (estimated from Theis recovery^59^) were used for a correlation with different structural zones (Zone-II and Zone-III) (Table 2). The result shows that Zone-II (1.46–2.84 m^2^/day) has relatively higher transmissivity than Zone-III (0.12–1.11 m^2^/day) due to the presence of different structural units. The higher the transmissivity value, the more prolific is the aquifer system. Unfortunately, the aquifer parameters T of Zone-I and Zone-IV were unavailable at this time due to other reasons.

**Table 2:** Aquifer properties and borehole locations in different structural zones (Ambaji Basin, NW India).

Well Location	Discharge ‘Q’ (m^2^/day)	Transmissivity ‘T’ (m^2^/day)	Zone	Structural features
Surela (B1)	100.22	N/A	I	Ductile Shear zones
Kengora (B2)	200.88	2.63	II	Ductile Shear zone with Fault/Fractures
Ajapur Mota-1 (B3)	137.13	1.79	II	
Ajapur Mota-2 (B4)	232.66	2.84	II	
Virampur (B5)	103.50	1.46	II	
Kanpura-1 (B6)	47.52	0.12	III	Brittle faults/fractures
Kanpura-2 (B7)	89.85	0.19	III	
Khapa EW I (B8)	155.52	0.39	III	
Hathidra EW (B9)	172.80	1.11	III	

## Discussion

### Overprinting of late brittle faults and fractures on Neoproterozoic foliations

As the foliations are near vertical (NW–SE trending in general) (Figs. 4, 5), the principal maximum stress component (σ_1_) must lie in the NE-SW quadrant (perpendicular to the plane of foliation), comparable with earlier studies^38^. The observed foliations are related to the Neoproterozoic (834–778 Ma) compression and shearing inferred from the published geochronology data^37,60^. Therefore, the Neoproterozoic compression resulted in the pervasive mylonitic foliations at higher temperatures (ductile deformation) widely observed in the field. From the cross-cutting relationships, it is evident that the brittle faults and fractures overprint the foliations. As the shear zones were developed in the Neoproterozoic (834–778 Ma) time, the brittle faults and fractures that overprint the shear fabrics must be post-Neoproterozoic deformation. The paleostress analysis indicates that NW–SE to NE-SW extension is responsible for the late brittle faults and fractures, which are sub-horizontal to sub-vertical to the general trend of the sub-vertical foliations (Figs. 4, 5, 9). As two distinct episodes of extensions events overprint the foliations, faults, and fractures related to both the events control the groundwater.

### Depth of weathered zone

**Figure 18:**
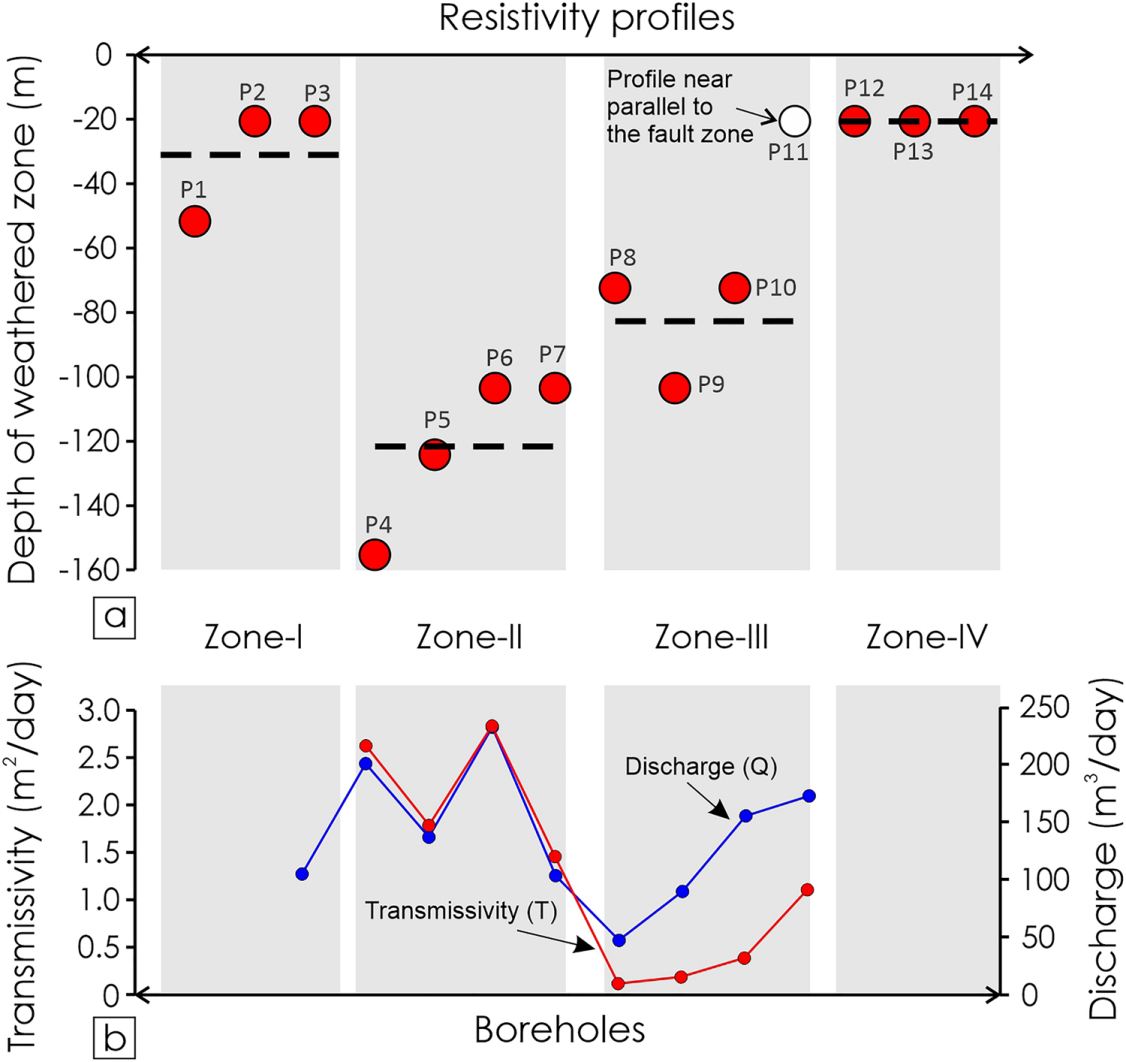
(**a**) Weathered zone depth in different zones estimated from resistivity profiles. Red dots denote the maximum weathered zone depth for different resistivity profiles. The dotted line marked the mean weathered zone depth. Profile-11 is an outlier in Zone-III as the profile is sub-parallel to the fault zone. Therefore, the weathered zone depth for Profile-11 is discarded. (**b**) Transmissivity (T) and discharge (Q) of different boreholes for different structural zones (Zone-I, Zone-II, and Zone-III).

Depth of the weathered zone is critical in hard rock terranes as this has direct control over the subsurface water resources. The control of weathering on the depth of the weathered zone is well studied^30,61–63^. However, the control of secondary tectonic structures like foliations, fractures, and faults is debatable. The depth of weathered zones is highest in Zone-II (~ 160 m) and gradually decreases as we move through Zone-III (~ 100 m) to Zone-IV (~ 20 m) (Fig. 18). Zone-IV is the domain with the lowest weathered zone depth (Fig. 18). Zone-I (~ 50 m) has a weathered zone depth higher than Zone-IV and lower than Zone-III (Fig. 18). As the precipitation for a particular year and the lithology are similar in all the zones (Figs. 1, 13), it seems to have insignificant control on the variability of the weathered zone depth.

The variability in the depth of the weathered zone in different zones (Figs. 13–16, 18) is likely to be controlled by the foliations, fractures, and faults. This is obvious that the fractures and faults add secondary porosity to the rocks and hence will help the water to percolate through and interact more with the rocks and increase the degree of weathering. Therefore, likely a thicker weathered zone will develop. If the fracture overprints a pre-existing planar pervasive feature like foliations, as in Zone-II, the chance of degree of weathering will be very high, resulting in a thick weathered zone compared to the zones having only faults and fractures. We proposed this is what is happening in the present study.

**Figure 19:**
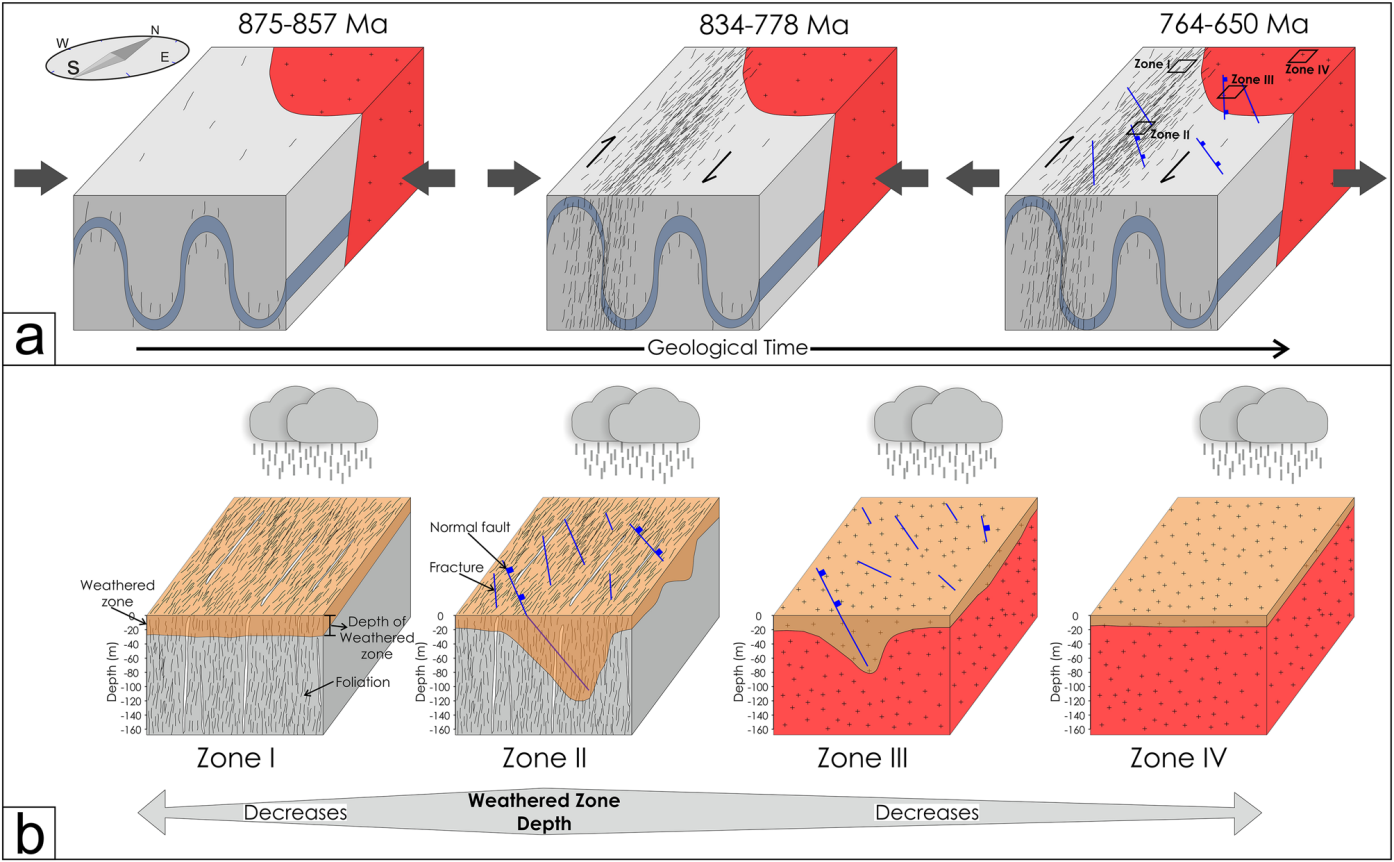
Conceptual model showing the different tectonic events and formation of respective weathered zone thickness. (**a**) Development of different planar fabrics over time. The early foliation was developed by the NE-SW compression between 875 and 857 Ma. The compression event is followed by an event of strike-slip shearing between 834 and 778 Ma. The last event, predominantly NE-SW extension, predominate the area between 764 and 650 Ma resulted the normal faults. Age data were compiled from Singh et al.^37^ and Tiwari and Biswal^60^. (**b**) Structurally controlled bedrock weathering in different zones resulted variability in the weathered zone thickness. Zone-II and Zone-IV have a maximum and minimum weathered zone thickness respectively.

The area was initially undergone a crustal thickening phase by folding between 875 and 857 Ma (Fig. 19). Then ductile shear zone development predominates, resulting in the sheared foliations between 834 and 778 Ma^37^ (Fig. 19). After that, the area has undergone a phase of extension marked by the late brittle fractures and faults between 764 and 650 Ma^37^ (Fig. 19). As there are two distinct and almost synchronous extension events overprinting one another and the foliation, individual fault planes cannot be attributed to a particular extension event from the paleostress results due to the lack of evidence to separate these two phases of extension events. Hence, the deduction of the role of each extension event is unfeasible. With the advent of time, weathering and erosion predominate the area. Zone-II, the domain in which the fractures overprint the foliation fabrics, has the thickest weathered zone (~ 160 m) compared to the other zones (Figs. 18, 19). As the two extension events are synchronous and indistinguishable, faults related to both the extension events have contributed to the weathered zone depth. This is because the fractures and faults add more secondary porosity to the zone having pre-existing foliation planes that facilitate the water to interact more with the rock and develop the thickest weathered zone. Hence, Zone-I with only foliations has a considerably thinner weathered zone than Zone-II, containing both fractures and foliations (Fig. 19). Similarly, in Zone-III and Zone-IV, the crystalline rock is massive, devoid of any pervasive foliations, therefore doesn’t easily facilitate the water to interact with the rocks. For this reason, Zone-IV which is massive and devoid of foliations, fractures, and faults, has the thinnest weathered zone compared to the other three zones. In comparison, Zone-III, a massive zone that contains late brittle faults and fractures, increases the surface area for water–rock interaction and creates a weathered zone thicker than Zone-IV (Fig. 19). In a nutshell, the zone with foliations overprinted by faults and fractures (Zone-I) facilitates water to interact more with the rocks resulting in the thickest weathered zone, and the zone devoid of foliations, fractures, and faults (Zone-IV) minimizes the area of water–rock interactions and hence has the thinnest weathered zone. For this reason, Zone-II is the most favorable site from the groundwater exploration point of view. Further, the preliminary yield test (PYT) of boreholes shows the variation in transmissivity in different structural zones (Table 2, Fig. 18). Higher transmissivity (1.46–2.84 m^2^/day) is witnessed in Zone-II whereas moderate to low transmissivity (0.12–1.11 m^2^/day) in the Zone-III areas (Fig. 18). This shows the good agreement between the different structural domains, weathered thickness, and respective groundwater storage in the subsurface. Moreover, as weathered bedrocks and soil plays a crucial role^30^, distinct structural zones (Zone-I to Zone-IV) with structurally controlled weathered zone thickness (Fig. 19) have different implications on “Critical Zone”, that accommodate varied critical biogeochemical processes.

## Conclusions

This study conclusively demonstrates the impact of foliations, fractures, and faults on the weathered zone depth by integrating structural, geophysical, and hydrogeological data sets. Moreover, the weathered zone thickness in these areas led to groundwater storage and possible recharge sites. Therefore, the effect of these structures on groundwater explorations cannot be neglected, and thus this study will improve the search methodology of groundwater exploration in hard rock terranes. As we demonstrate the role of tectonic structures on weathered zone thickness and groundwater storage, this study also has implications on “Critical Zone”.

## Supplementary Information

Supplementary Information 1.

Supplementary Information 2.

Supplementary Information 3.

Supplementary Information 4.

## Data Availability

The datasets used and/or analysed during the current study available from the corresponding author on reasonable request.
