# Supplementary material for: Structural controls on bedrock weathering in crystalline basement terranes and its implications on groundwater resources
Source: Sci Rep. 2022 Jul 12;12:11815. doi: 10.1038/s41598-022-15889-x (PMC9276672; doi:10.1038/s41598-022-15889-x)

**Appendix 1.** Forward and inversion parameters.

| **Parameter** | **Description** |
| --- | --- |
| Forward Solver | Finite element method |
| Electrode array | Gradient/Schlumberger |
| Unit spacing (m) | 10/5 |
| No. of observed data | 700-750 |
| No. of triangle | ~3000 |
| $\lambda_{1}$ (initial value) | Computed based on standard deviation of measured data |
| $\lambda_{1}$ (Minimum value) | 0.01 |
| $\lambda_{1}$(damping factor) | 0.55 |
| Maximum No. of iteration | 10 |
| Stopping criteria | Root mean square (RMS) will no longer reduced |

**Appendix 2**. Depth to water level during Pre-monsoon season (May 2017).


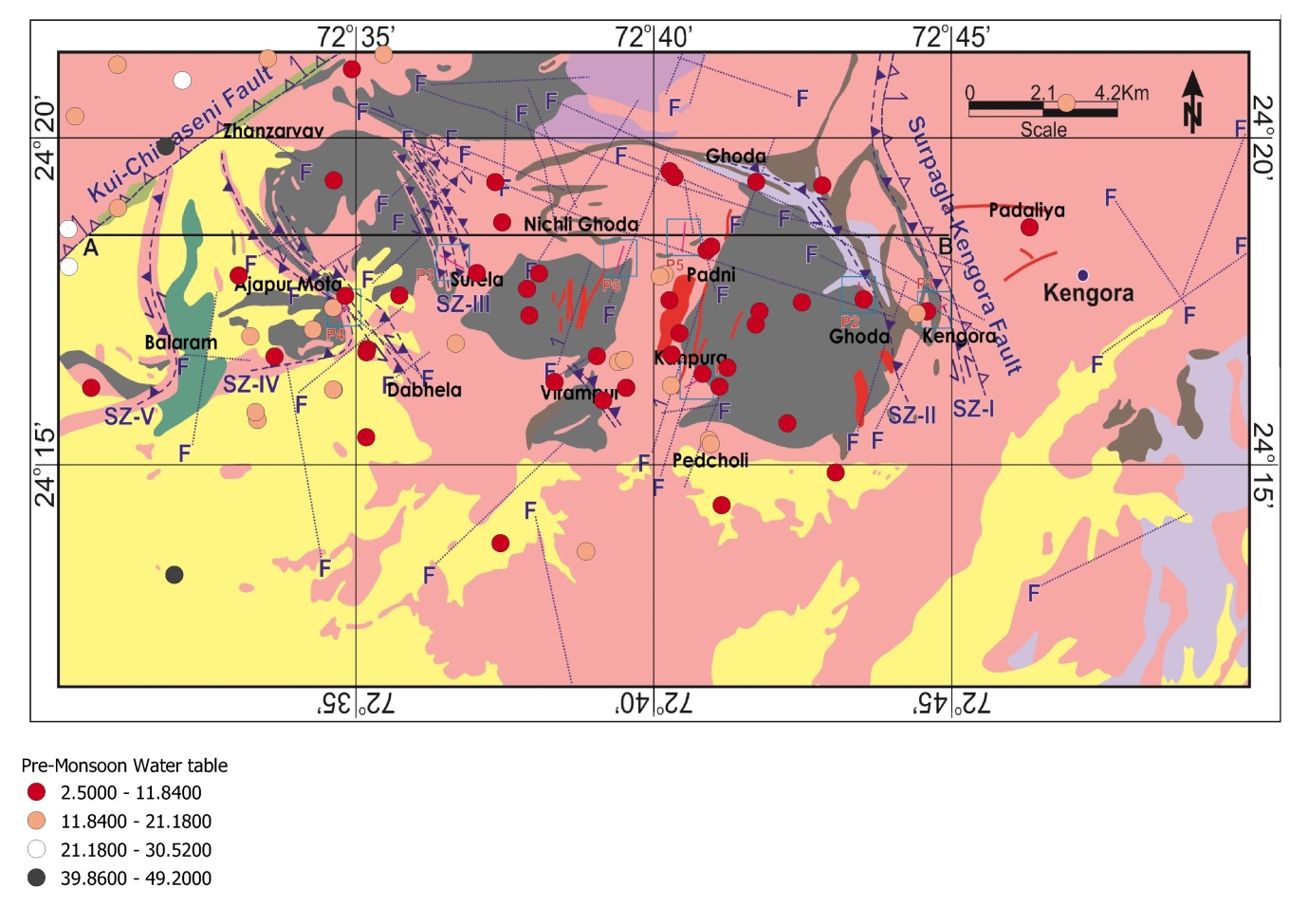


**Appendix 3**. Depth to water level during Post-monsoon season (December 2017).


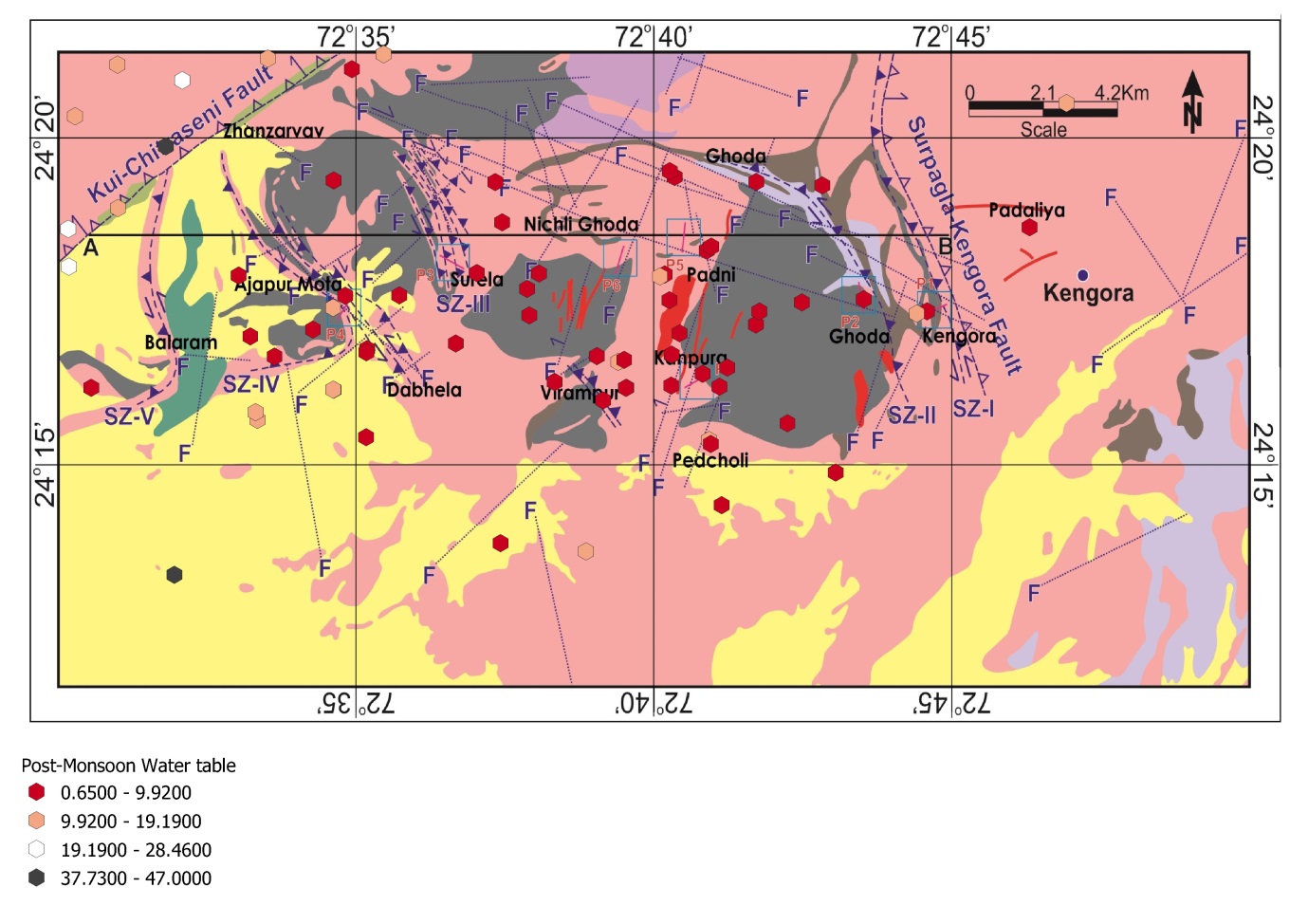

Supplement: Supplementary file 1 — Supplementary Information 1. [file 41598_2022_15889_MOESM1_ESM.docx]
